# The NFIB/CARM1 partnership is a driver in preclinical models of small cell lung cancer

**DOI:** 10.1038/s41467-023-35864-y

**Published:** 2023-01-23

**Authors:** Guozhen Gao, Simone Hausmann, Natasha M. Flores, Ana Morales Benitez, Jianjun Shen, Xiaojie Yang, Maria D. Person, Sitaram Gayatri, Donghang Cheng, Yue Lu, Bin Liu, Pawel K. Mazur, Mark T. Bedford

**Affiliations:** 1 Department of Epigenetics and Molecular Carcinogenesis, The University of Texas MD Anderson Cancer Center, Houston, TX 77030 USA; 2 Department of Experimental Radiation Oncology, The University of Texas MD Anderson Cancer Center, Houston, TX 77030 USA; 3 Center for Biomedical Research Support, The University of Texas at Austin, Austin, TX 78712 USA; 4 Department of Pediatrics, The University of Texas MD Anderson Cancer Center, Houston, TX 77030 USA; 5 Present Address: Evozyne Inc., Chicago, IL 60614 USA

**Keywords:** Methylation, Cancer genetics

## Abstract

The coactivator associated arginine methyltransferase (CARM1) promotes transcription, as its name implies. It does so by modifying histones and chromatin bound proteins. We identified nuclear factor I B (NFIB) as a CARM1 substrate and show that this transcription factor utilizes CARM1 as a coactivator. Biochemical studies reveal that tripartite motif 29 (TRIM29) is an effector molecule for methylated NFIB. Importantly, NFIB harbors both oncogenic and metastatic activities, and is often overexpressed in small cell lung cancer (SCLC). Here, we explore the possibility that CARM1 methylation of NFIB is important for its transforming activity. Using a SCLC mouse model, we show that both CARM1 and the CARM1 methylation site on NFIB are critical for the rapid onset of SCLC. Furthermore, CARM1 and methylated NFIB are responsible for maintaining similar open chromatin states in tumors. Together, these findings suggest that CARM1 might be a therapeutic target for SCLC.

## Abstract (web summary)

Protein arginine methylation can contribute to tumor progression. Here the authors show that protein arginine methyltransferase, CARM1, methylates transcription factor NFIB to promote the growth of small cell lung cancers.

## Introduction

Protein arginine methylation is a pervasive posttranslational modification (PTM) that plays an important role in cell signaling, but when deregulated, can be associated with different diseases. Indeed, the overexpression of protein arginine methyltransferases (PRMTs) is frequently seen in human cancers, and is generally associated with poor clinical outcome^1,2^, which has prompted the development of PRMT small molecule inhibitors^3^. There are nine enzymes in the PRMT family: PRMT1-9; PRMT4 is also referred to as CARM1. CARM1 together with PRMT1, 2, 3, 6, & 8, are classified as type I PRMTs that catalyze the deposition of asymmetric dimethylarginine (ADMA), while PRMT5 and 9 are grouped together as type II PRMTs and are responsible for symmetric dimethylarginine (SDMA)^4^.

CARM1 was the first PRMT to be implicated in transcriptional regulation through its ability to be recruited by nuclear receptors and then methylate histone H3^5^. Subsequent studies have sought to unravel the biological roles of CARM1 by screening for its substrates using protein arrays^6^, and by the direct testing of candidate substrates^7^, small-pool screening^8^ and mass spectrometry approaches^9,10^. A host of CARM1 substrates have been identified, which clearly places this enzyme in a central position for coactivating transcription. It methylates and regulates the activity of other coactivators like p300/CBP^11^ and the H3K4 methyltransferases KMT2C and KMT2D^9,10^. It also stabilizes promoter/enhancer looping by modifying MED12 in the Mediator complex^9,12^. CARM1 modulates chromatin-remodeling by targeting Pontin^13^. Finally, CARM1 directly methylates a number of transcription factors including PAX7^14^, SOX2^15^, RUNX^16^, and YY^17^. Mechanistically, a CARM1 methylated substrate is often able to recruit an effector molecule, or “reader”, to the newly created methyl-motif. One such effector is TDRD3^18^, which binds both the histone H3R17me2a and the MED12-R1899me2a marks^9,19^, and in-turn recruits TOP3B to chromatin to resolve R-loops and untangle RNA structures^20^. TDRD3 harbors a Tudor domain for the purpose of reading the ADMA marks deposited by CARM1 and PRMT1. Other Tudor domain-containing proteins like SND1, SMN, SPF30 and TDRD1, all selectively interact with SDMA marked motifs^21^. It is likely that additional methylarginine effector proteins exist.

To expand our understanding of how CARM1 regulates cellular processes, we performed a focused search of additional substrates of this enzyme and identified the nuclear factor I (NFI) family of transcription factors. The NFI family has four members: NFIA, NFIB, NFIC, and NFIX^22^, and all four harbor a conserved motif that can be modified by CARM1. Importantly, both *Carm1* and *Nfib* null mice display very similar lung hyperplasia that causes death just after birth due to respiratory defects^23–25^, possibly indicative of a mechanistic link between CARM1 and NFIB in the lung. NFIB was reported as an oncogene that promotes metastasis of small cell lung cancer (SCLC) by increasing the chromatin accessibility in gene distal regions^26–28^. High levels of NFIB are associated with human SCLC metastases and poor overall survival^28^. Like NFIB, CARM1 is often overexpressed in human cancers, including lung cancer^1,29^. Furthermore, in a mouse model of overexpression, elevated CARM1 levels in Keratin 5 (K5) expressing tissues (skin and the mammary gland) present with a high incidence of spontaneous tumors^30^. These findings suggest that CARM1 inhibitors may have therapeutic value, and such compounds have been developed by Pharma^31,32^ and in an academic setting^33^. CARM1 inhibitors display anti-tumor activity, in a pre-clinical setting, against multiple myeloma^31,32^, acute myeloid leukemia^34^, breast cancer^33^ and diffuse large B-cell lymphoma^35^.

The recent study showing that CARM1 inhibition is a vulnerability for CREBBP/EP300 mutations carrying lymphomas^35^, prompted us to investigate if other tumor types that are driven by CARM1 substrates may also be responsive to CARM1 inhibitors. Here, we explored the consequence of NFIB methylation, and show that the TRIM29 protein interacts with NFIB in a CARM1-dependent manner. The NFIB methylation site (R388) is important for promoting the transcription of its target genes, and as an effector of this mark, TRIM29 functions as a transcriptional coactivator. These cell-based studies encouraged us to investigate the importance of NFIB methylation by CARM1 in vivo. Using a genetically engineered mouse model (GEMM) for SCLC, we find that both *Carm1* conditional loss and *Nfib*^R388K^ knock-in results in a significant increase in survival, and the tumors derived from these two models display very similar patterns of open chromatin. Furthermore, SCLC PDX models are sensitive to CARM1 inhibitor treatment. These studies suggest that tumors that are driven by NFIB amplification are vulnerable to the targeting of CARM1 activity.

## Results

### NFIB is a CARM1 substrate

**Fig. 1:**
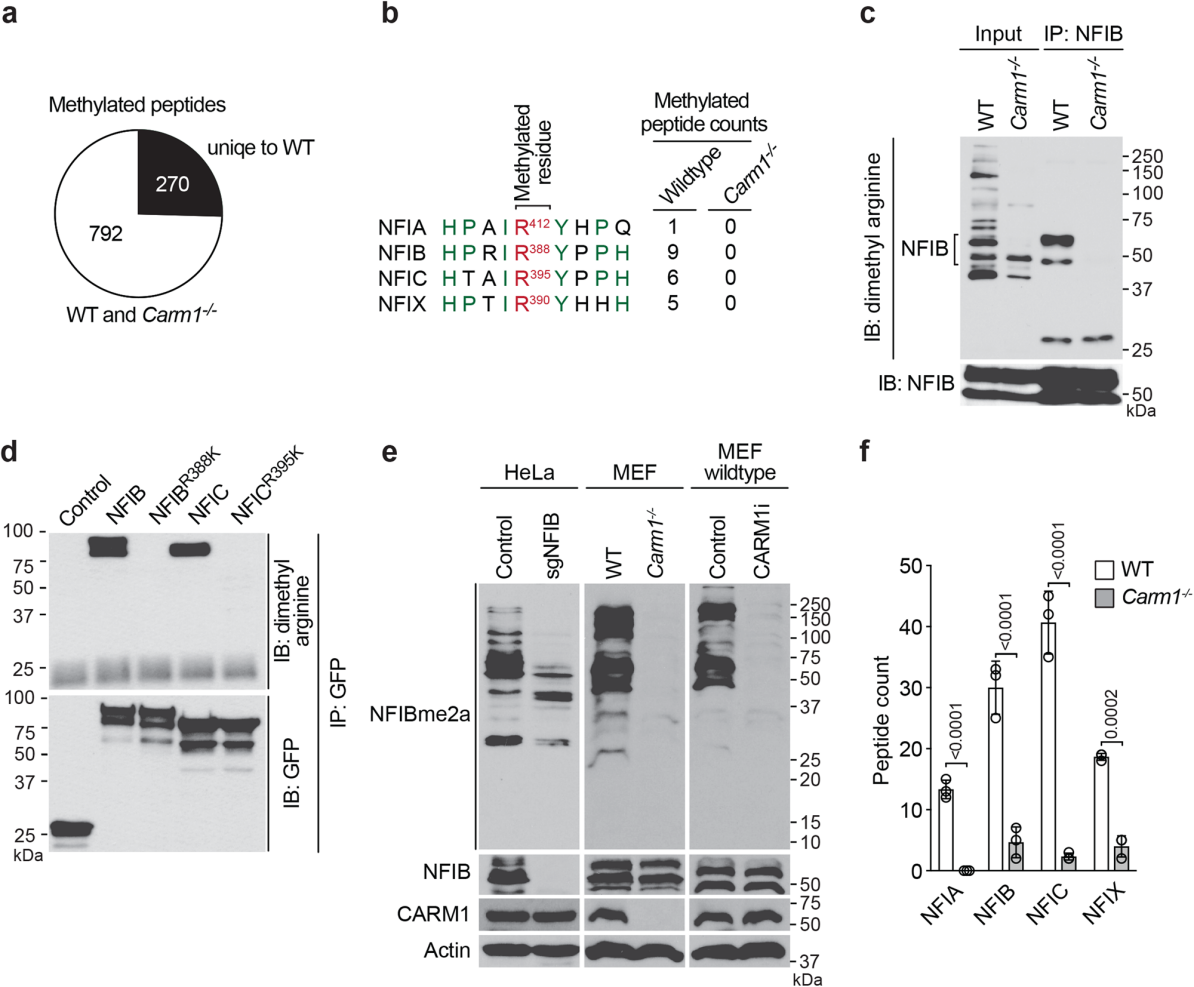
Identification of NFIB as a CARM1 substrate. **a** ADMA-containing proteins were immunoprecipitated from CARM1 wild-type (WT) or knockout (*Carm1*^*−/−*^) MEFs, and identified by LC-MS/MS. **b** Identified peptides from the nuclear factor I family. **c** NFIB protein was immunoprecipitated from either CARM1 WT or KO MEFs, and immunoblotted with antibodies against ADMA (D4H5) or NFIB. **d** GFP vector control, GFP-NFIB-WT or -R388K, GFP-NFIC-WT or -R395K constructs were transfected into HeLa cells. GFP-tagged proteins were immunoprecipitated and immunoblotted with antibodies against ADMA (D4H5) or GFP. **e** Validation of NFIB methylation using the NFIBme2a antibody. Total cell lysates were analyzed from NFIB WT/KO HeLa cells, CARM1 WT/KO MEFs and WT MEFs treated with CARM1 inhibitor (CARM1i, TP-064). Actin shown as a loading control. **f** IP-MS was performed, in triplicate, on cell lysates from WT or *Carm1*^*−/−*^ MEFs using the NFIBme2a antibody. Exclusive spectrum counts for all the four NFI family proteins were analyzed and plotted. Data present in mean ± SEM*; P* values determined by two-tailed student’s t-test. All blots are shown as representative data from three independent experiments.

To identify substrates for CARM1, we immunoprecipitated ADMA-containing peptides using previously reported ADMA antibodies^36^ from proteolytically digested CARM1 wild-type (WT) and knockout (KO) mouse embryonic fibroblasts (MEFs). Both the antibodies (D4H5 and D6A8) used for the enrichment of ADMA methylated peptides have been shown to recognize a subset of CARM1 substrates, but they also recognize PRMT1 substrates^36^. Thus, the use of CARM1 WT and KO MEFs in this experiment will help us identify the CARM1-specific substrates. The ADMA-enriched peptides were then identified by LC-MS/MS. A total of 1062 peptides that contain di-methylated arginine were cataloged (Supplementary Data S1A). Among them, 270 peptides were identified from CARM1 WT, but not KO MEFs (Fig. 1a), suggesting that these are sites of CARM1-mediated methylation.

Notably, all the nuclear factor I (NFI) family members (NFIA, NFIB, NFIC and NFIX) were identified as methylated in CARM1 WT MEFs only, and they harbor a conserved amino acid sequence around the methylated arginine site (Fig. 1b). The motif is proline-rich, which is indicative of a CARM1 methylation motif and consistent with the reported recognition motif for the D4H5 antibody used for the peptide-enrichment step^10^. The NFI peptides constitute about 8% of total peptides unique to CARM1 WT MEFs, indicating that they are dominant targets for CARM1. We are particularly interested in the methylation of NFIB, since both *Nfib* and *Carm1* knockout mice die perinatally, due to a similar deficiency in lung development^24,25,37,38^. To confirm that NFIB is methylated by CARM1 in the cells, we immunoprecipitated (IPed) NFIB from both CARM1 WT MEFs and CARM1 KO MEFs, and detected the methylation of NFIB by immunoblotting with the specific ADMA antibody (D4H5) (Fig. 1c). The ADMA antibody only detected IPed NFIB from CARM1 WT, but not CARM1 KO MEFs. Furthermore, we then made GFP fusions of NFIB or NFIB^R388K^ (where the arginine methylation site is mutated to lysine) and transfected them into HeLa cells. The GFP-tagged NFIB proteins were then IPed for the detection of ADMA by Western (Fig. 1d). Methylation on wild-type, but not on mutated NFIB, can be observed. Similarly, methylation of NFIC is abolished when R395 is mutated to lysine.

We also generated our own methyl-specific antibody that recognizes the NFIB^R388me2a^ motif (Supplementary Fig. 1a) and produced NFIB KO HeLa cells to help characterize this antibody (Supplementary Fig. 1b). This purified antibody (NFIBme2a) was tested on total cell lysates from NFIB WT and KO HeLa cells, CARM1 WT and KO MEFs, as well as WT MEFs treated with vehicle or a specific CARM1 inhibitor^31^ (TP-064). This NFIBme2a antibody is totally CARM1-dependent as it does not recognize any proteins when CARM1 is absent or inhibited (Fig. 1e). The Western analysis of the NFIB knockout cells reveals that the NFIBme2a antibody does recognize additional CARM1 methylated proteins, apart from NFIB, and these might be other NFI family members (Fig. 1e). Indeed, mass spec. analysis was performed in triplicate on CARM1 WT and KO MEFs, using our own methyl-specific antibody, and we identified strong enrichment of the NFI family members (Fig. 1f), as well as a number of additional proteins that are putative CARM1 substrates (Supplementary Data S1B).

### CARM1 is a transcriptional coactivator for NFIB

NFIB is a transcription factor that governs the expression of numerous genes critical for SCLC differentiation, hair follicle stem cell behavior, androgen receptor signaling, megakaryocyte cell maturation and neuron development^39^. We were interested in determining whether the methylation of NFIB by CARM1 is a way of promoting its transcriptional activity. To identify direct NFIB target genes, we performed chromatin immunoprecipitation followed by sequencing (ChIP-seq) to detect the distributions of NFIB binding sites across the genome of HeLa cells. ChIP-seq was performed using two independent NFIB antibodies, and also using a GFP antibody on a HeLa cell line that stably expresses GFP-NFIB (Supplementary Fig. 1c). By comparing all 3 sets of ChIP-seq data, we found 1696 high-confident loci were directly bound by NFIB, which we refer to as NFIB^YYY^ (YYY = yes for all three Abs) (Supplementary Fig. 1d). We also performed a ChIP-seq experiment using an antibody that recognizes the histone H3R17me2a mark, which CARM1 deposits. Although this H3R17me2 antibody recognizes the methylation site on H3, it also cross-reacts with many other CARM1 substrates^8^, and methylated NFIB itself (Supplementary Fig. 1e). Thus, the H3R17me2a ChIP peak denotes regions of CARM1 activity, and not necessarily the H3R17me2a mark itself. We found that roughly one third (523) of the NFIB^YYY^ peaks overlap with sites that harbor CARM1 activity (Supplementary Fig. 1f and Supplementary Data S2), and represent NFIB-bound loci that are candidates for CARM1 regulation. We selected six NFIB-bound loci from this set of 523 peaks and validated this binding by ChIP-qPCR (Supplementary Fig. 1g). To test whether methylation of NFIB affects transcription regulation, we compared the mRNA levels of these selected genes upon the overexpression of wild-type NFIB (NFIB-WT) or the methylation-deficient mutant (NFIB-R388K). Generally, overexpression of NFIB-WT significantly increased the mRNA levels of the selected genes, while overexpression of NFIB-R388K did not promote transcription (Supplementary Fig. 1h). Consistent with this finding, inhibition of NFIB methylation by the CARM1 inhibitor, TP-064, also significantly inhibited the expression of all the selected NFIB target genes, except FERMT1 (Supplementary Fig. 1i). These results indicate that the methylation of NFIB by CARM1 plays an important role in regulating the gene expression program driven by this transcription factor.

### TRIM29 is an effector molecule for methylated NFIB

**Fig. 2:**
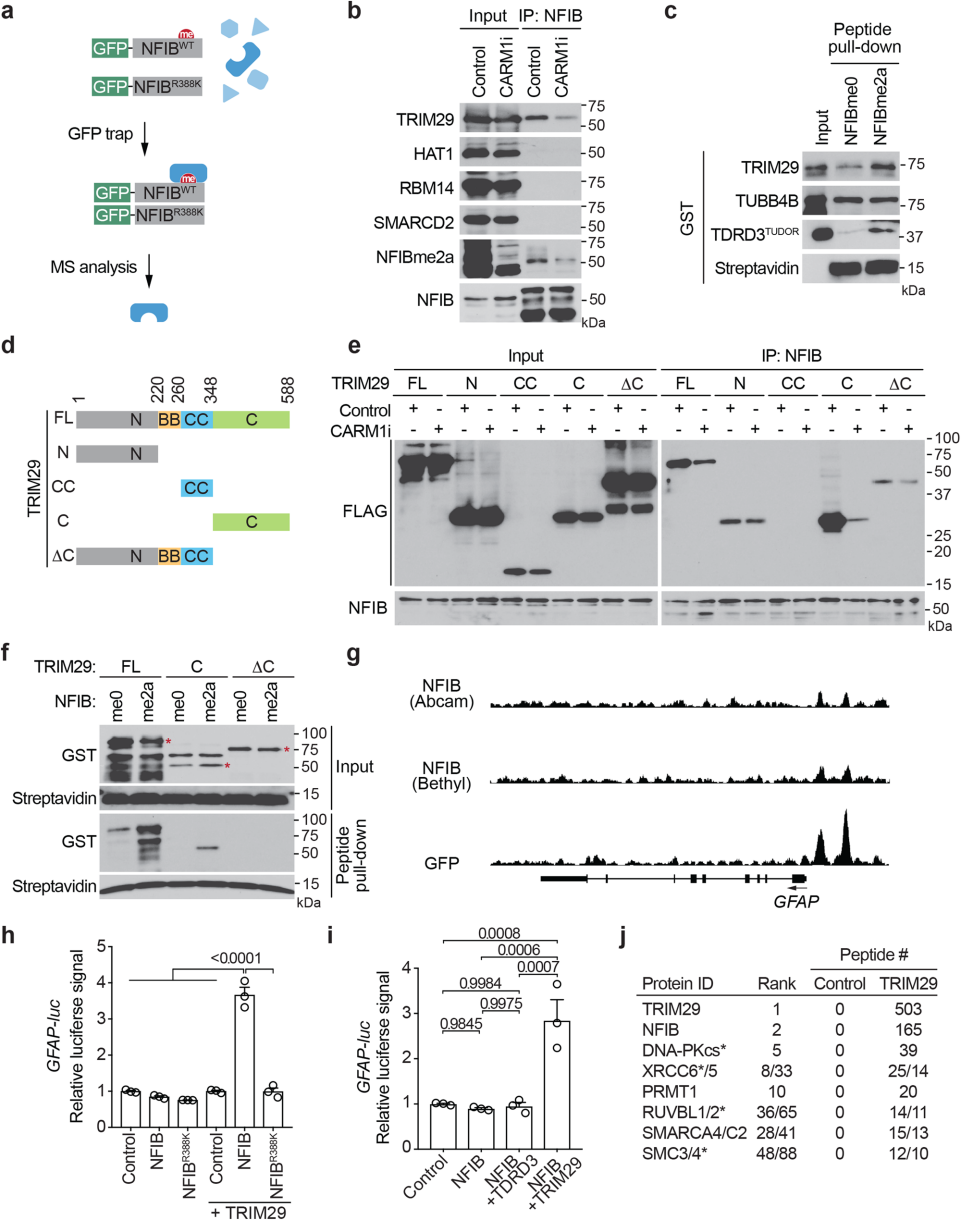
TRIM29 is an effector for methylated NFIB dentification. **a** Workflow of the screening for proteins that bind wild-type but not mutant NFIB. **b** HEK293T cells were transfected with indicated transcription regulators. The cells were cultured in the presence or absence of CARM1 inhibitor. The NFIB protein complex was immunoprecipitated and the association of these candidates was tested by Western blot, using an anti-FLAG antibody. **c** GST-tagged full-length TRIM29 was incubated with biotin-labeled unmethylated or methylated NFIB peptides. Peptide pull-down was performed using streptavidin-conjugated beads. **d** Scheme showing the domains of TRIM29 that were fused to FLAG and GST for the experiments performed in (**e**) and (**f**). **e** Full length and truncated TRIM29 constructs were transfected into HEK293T cells. The cells were cultured in the presence or absence of CARM1 inhibitor. NFIB was IPed and the interaction of these TRIM29 mutants was tested by Western blot, using an anti-FLAG antibody. **f** GST-tagged full-length and truncated TRIM29 were incubated with biotin-labeled unmethylated or methylated NFIB peptides. Peptide pull-down was performed using streptavidin-conjugated beads. **g** ChIP-seq tracks showing the distribution of NFIB in promoter regions of GFAP gene. Three different antibodies were used to generate the tracks: an Abcam antibody, a Bethyl antibody, and a GST antibody that detects ectopically expressed GFP-NFIB. **h** HEK293T cells were co-transfected with GFAP-luc, together with GFP control construct, NFIB-WT, NFIB-R388K, TRIM29, TRIM29 plus NFIB-WT, or TRIM29 plus NFIB-R388K. The luciferase activities were detected by dual luciferase assay. The relative luciferase activities are normalized to the GFP control. Experiments were performed in biological triplicate. Data present in mean ± *SEM*; *P* values determined by two-tailed student’s t-test. **i** HEK293T cells were co-transfected with GFAP-luc, together with GFP control construct, NFIB, NFIB plus TDRD3, or NFIB plus TRIM29. The luciferase activities were detected by dual luciferase assay. The relative luciferase activities are normalized to the GFP control. Experiments were performed in triplicate. Data present in mean ± SEM; *P* values determined by two-tailed student’s t-test. **j** TRIM29-interacting proteins are identified by GFP trap in H69 SCLC cell line, followed by MS analysis as in (**a**). List of high-confidence candidate interacting proteins from MS analysis of GFP-tagged TRIM29 and GFP (control). Asterisk denotes TRIM29-binding proteins that have previously been identified by MS^42^. All blots are shown as representative data from three independent experiments.

As a transcription factor, NFIB harbors DNA-binding domains towards its N-terminus, and the C-terminal region carries the transactivation and repression activities^40^. The CARM1 methylation site on NFIB (R388) is located in the C-terminal transcription modulation domain, and thus this methylation will likely not impact the DNA-binding properties of NFIB. In many cases, the posttranslational modification of TFs regulates their activator/repressor functions by facilitating the recruitment of effector proteins that co-regulate transcription. We thus sought to identify a “reader” for the NFIB methylation site. To do so, we ectopically expressed GFP-tagged NFIB-WT or its R388K mutant version in HeLa cells. We then purified the respective protein complexes using the GFP-Trap affinity approach and identified the interacting proteins by proteolytic digestion and LC-MS/MS (Fig. 2a and Supplementary Data S3). We were interested in identifying proteins that bound GFP-NFIB but not GFP-NFIB^R388K^, as these will be the candidate effector proteins for the CARM1 methylation site. We selected 15 proteins that were identified by two or more peptides in the GFP-NFIB complex and displayed zero peptides in the GFP-NFIB^R388K^ complex. Most of the selected proteins had reported functions related to transcriptional regulators. These effector candidates were cloned into an expression vector with a tag and overexpressed in HeLa cells (in a few cases endogenous proteins were directly detected using commercial antibodies), and the cells were then treated with DMSO or CARM1 inhibitor. Proteins interacting with NFIB were co-immunoprecipitated and examined for FLAG tag signal (Fig. 2b). TRIM29 was shown to interact with NFIB in a CARM1-dependent manner. This methylation-dependent interaction was further validated by an in vitro peptide pull-down assay, where GST-tagged TRIM29 preferentially binds to methylated NFIB peptides (Fig. 2c), demonstrating that this is a direct interaction. TDRD3 is a methylation reader that binds to many proteins marked with ADMA, including histones H3 and H4, RNA polymerase II, and MED12^9,19,41^. Here, we show that TDRD3 can also bind to the CARM1 methylation site on NFIB, and they can be co-IPed (Fig. 2c and Supplementary Fig. 2a). Thus, the methylated form of NFIB may be coregulated by either TRIM29 or TDRD3, which was further investigated.

We next mapped the region of TRIM29 that directly interacts with methylated NFIB. To do so, we generated four truncated forms of TRIM29 (Fig. 2d) and subjected these FLAG-tagged proteins to a co-IP with endogenous NFIB. For this experiment, HeLa cells were also treated with CARM1 inhibitor. We observed that both the N-terminal domain and the C-terminal domain of TRIM29 interact with NFIB (Fig. 2e). When NFIB methylation is inhibited, the interaction between the C-terminus and NFIB is significantly reduced, while interaction between the N-terminus and NFIB remained unchanged, suggesting that the C-terminal domain of TRIM29 is responsible for the methylation-dependent interaction. To confirm this, a peptide pull-down assay was performed using GST fusion of full-length TRIM29, as well as fusions of the C-terminal domain only, and a fusion of TRIM29 without its C-terminus (Fig. 2d). Clearly, the C-terminal domain specifically interacts directly with methylated NFIB (Fig. 2f). Using a co-IP assay, we also show that endogenous NFIB and TRIM29 interact, and that this interaction is prevented by CARM1 inhibitors (Supplementary Fig. 2b).

The C-terminal domain of TRIM29 was reported to mediate interactions with many proteins^42^. This region is an orphan domain that has no homologous regions in other proteins. To further characterize TRIM29 as an arginine methylation reader, we incubated the C-terminal domain of TRIM29 with a methylated GAR motif from fibrillarin. TRIM29 interacted strongly with the fibrillarin GAR motif that has the ADMA marks, but not the SDMA form (Supplementary Fig. 2c). As expected, the Tudor domain of TDRD3 showed stronger binding to ADMA marks, while the Tudor domain of SMN showed stronger binding to SDMA marks. Thus, TRIM29 likely binds additional methylated proteins apart from just NFIB.

### TRIM29 promotes the transcription of NFIB target genes

Effectors that specifically bind to arginine methylated transcription regulators can shape the downstream transcription program^13,19,43^. GFAP is a well-studied target gene of both NFIA and NFIB in astrocytes^44–46^, and a direct NFIB target identified by our ChIP-seq. Indeed, both NFIB antibodies (Abcam and Bethyl) display GFAP promoter peaks, which are elevated in ectopic GFP-NFIB expression (Fig. 2g). To establish if TRIM29 can coactivate the expression of NFIB regulated GFAP, we performed an in vitro luciferase reporter assay. We found that TRIM29 can significantly promote transcription driven off the GFAP promoter. This coactivator activity is seen when wild-type NFIB is overexpressed with TRIM29, but not when the methylation deficient NFIB mutant is overexpressed (Fig. 2h). Next, we compared the coactivator activity of TRIM29 and TDRD3, as we have shown that both these proteins can function as readers of the methylated NFIB peptide (Fig. 2c). Using the GFAP luciferase reporter assay, we see that TRIM29, but not TDRD3, functions with NFIB to coactivate expression of this promoter (Fig. 2i and Supplementary Fig. 2d). We thus focused our attention on TRIM29 as a novel effector molecule for methylated NFIB. Next, we confirmed TRIM29 protein expression in the cell lines used in this study (Supplementary Fig. 2e) and observed that expression of the majority of the identified NFIB-regulated genes (see Supplementary Fig. 1g) is promoted upon overexpression of TRIM29 (Supplementary Fig. 2f).

Overexpression of NFIB is correlated with advanced tumor stages and metastasis in small cell lung cancers^27,28^ therefore, we hypothesize that methylation of NFIB may also regulate the progression of SCLC. Since the mechanistic studies described above were performed in HeLa and HEK293T cells, we sought to confirm the NFIB/TRIM29 interaction in SCLC cells. To that end, we performed the GFP-trap experiment with GFP-TRIM29 in H69 SCLC cell line and found that the top-ranked binding protein is NFIB (Fig. 2j and Supplementary Data S4). Thus, reciprocal GFP-trap experiments in HeLa and H69 cell lines identified the NFIB/TRIM29 interaction (Fig. 2a, j).

### CARM1-mediated methylation of NFIB is required for tumor growth of SCLC in vivo

**Fig. 3:**
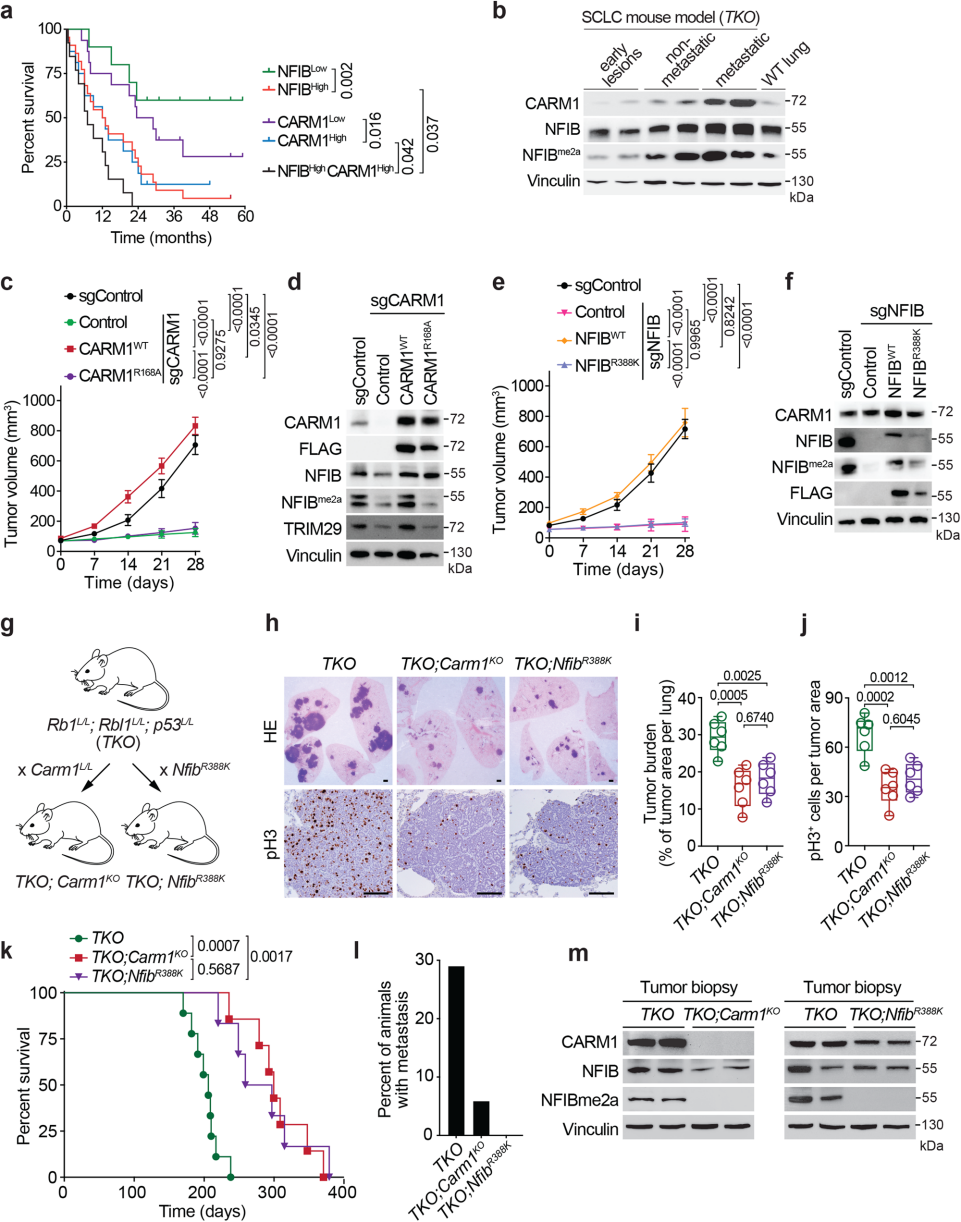
CARM1-mediated methylation of NFIB at K388 promotes SCLC pathogenesis. **a** High expression of CARM1 and NFIB predicts poor SCLC patient survival. Analysis of SCLC tissue array IHC for CARM1 and NFIB. *P* values by Log-rank test, *n* = 32 (16 ♂ / 16 ♀, no sex difference noted). **b** Immunoblot analysis of CARM1, NFIB and NFIB^R388me2a^ in three independent and representative tissue biopsies lysates (of six analyzed) from SCLC mouse model (*TKO*) at early and late stages of tumorigenesis with non-metastatic and metastatic disease. **c** H69 SCLC cells xenograft growth in NSG mice modified to express sgRNA CARM1 or sgRNA control and overexpressing WT or catalytically deficient R168A CARM1 (*n* = 5; *P* values by two-way ANOVA with Tukey’s testing for multiple comparisons, data represent mean ± SEM). **d** Immunoblots with indicated antibodies of cell lysates as in (**c**). **e** Complementation of sgRNA NFIB depleted H69 SCLC cells with WT or R388K mutant NFIB shows that NFIB methylation is required for SCLC cell growth in vivo (*n* = 5; *P* values determined as in (**c**)). sgControl as in (**c**). **f** Immunoblots with indicated antibodies of cell lysates as in (**e**). **g** Schematic of the *Rb1; Rbl2; Trp53* (*TKO*) mouse SCLC models harboring either a conditional *Carm1*^*L/L*^ knockout allele or a *Nfib* R388K mutant. **h** Representative HE and IHC of phospho-H3 staining, scale bars, 100 µm. **i** Quantification of tumor burden in indicated mutant mice (*n* = 6, balanced ♂/♀, no sex difference noted). *P* values by two-way ANOVA with Tukey’s testing for multiple comparisons; box plots, the line indicates the median, the box marks the 75th and 25th percentiles and the whiskers indicate the minimum and maximum values. **j** Quantification of proliferation marker (phospho-H3^+^ cells) in indicated mutant mice as in (**i**) (*n* = 6). **k** Kaplan–Meier survival curves of *TKO* control (*n* = 9, median survival 193d) and *TKO;Carm1*^*KO*^ (*n* = 7, 297d) *and TKO;Nfib*^*R388K*^ mutant mice (*n* = 6, 288d). *P* values by Log-rank test, balanced ♂/♀, no sex difference noted. **l** Liver metastasis incidence in the indicated mouse models as in (**i**). **m** Immunoblots with indicated antibodies of two independent and representative tumor biopsy lysates from indicated mutant mice. In all panels, Vinculin is a loading control.

We examined the CARM1-NFIB regulatory module in SCLC by immunostaining human tumor samples for CARM1, NFIB and NFIBme2a. We found that high levels of CARM1 and NFIB are independently predictors of poor patient survival (Fig. 3a) and a combination of CARM1^high^/NFIB^high^ predicts worse survival (Fig. 3a). Next, we analyzed tumors harvested from *TKO* mouse model of SCLC which carries conditional knockout of three tumor suppressors (*Rb1, Rbl2, Trp53*)^47^ commonly mutated in human cancer. We observed increased levels of CARM1, NFIB, and NFIBme2a as cancer progresses into malignant disease (Fig. 3b). In addition, we noted that NFIB expression correlates with human SCLC cell lines sensitivity to CARM1 inhibitor (CARM1i, TP-064) (Supplementary Fig. 3a). To investigate the importance of CARM1’s enzymatic activity in driving SCLC development, we performed a xenograft using modified H69 (Fig. 3c–f) and CORL47 (Supplementary Fig. 3b, c) cells. These cells harbored a CRISPR-mediated knockout of CARM1 and were rescued with either WT or enzyme-dead (R168A) CARM1. Tumor volume was dramatically impaired when CARM1 was ablated, and this phenotype was not rescued when the KO cells were complemented with mutant CARM1 (Fig. 3c, d and Supplementary Fig. 3b). Our results implicate CARM1 methylation of NFIB in SCLC tumorigenesis. Thus, we wanted to independently test the role of NFIB R388 methylation in the regulation of cancer expansion. Depletion of NFIB led to attenuated growth of SCLC H69 cells in vivo (Fig. 3e, f). Complementation with wild-type NFIB, but not NFIB harboring an R388K substitution, was able to fully restore cancerous growth in vivo (Fig. 3e, f and Supplementary Fig. 3c). We further confirmed that CARM1i phenocopies genetic ablation of CARM1 and NFIB methylation in H69 and CORL47 cells and that inhibitor does not exert an unspecific effect on cancer cells growth (Supplementary Fig. 3d, e). Similar to genetic or pharmacological inhibition of CARM1 and ablation of NFIB methylation, we observed reduced tumorigenic capacity of SCLC cells upon TRIM29 deletion (Supplementary Fig. 3f, g). Together, these data argue that CARM1 regulates SCLC tumor growth via methylation of NFIB R388 and the subsequent recruitment of the methylation reader TRIM29.

### The CARM1/NFIB partnership promotes cancer in SCLC GEMMs

To directly test the role of the CARM1-NFIB axis in SCLC pathogenesis, we generated a *Nfib*^*R388K*^ mutant mouse model using a CRISPR/Cas9 strategy. The donor DNA for the homologous recombination-driven mutation was designed to replace R^388^ with a lysine residue, and at the same time, disrupt an EcoRV site in the *Nfib* gene (Supplementary Fig. 3h). The loss of the EcoRV site made for efficient genotyping (Supplementary Fig. 3i). *Nfib*^*R388K*^ homozygous mice were viable and displayed no overt phenotypes. To further validate the establishment of a mutant NFIB mouse model, we immunoprecipitated NFIB from lung and spleen tissue of wild-type or *Nfib*^*R388K*^ homozygous mice and performed Western analysis using the anti-NFIBme2a antibody we developed (Fig. 1e). NFIB IPed from wild-type mice, but not *Nfib*^*R388K*^ homozygous mice, can be detected with the anti-NFIBme2a antibody, thus confirming that the R388 site is mutated in the mouse model (Supplementary Fig. 3j). We then crossed the *Nfib*^*R388K*^ mouse with the TKO GEMM of SCLC to obtained *TKO;Nfib*^*R388K*^ mice (Fig. 3g). In addition, we have previously generated a conditional knockout allele for CARM1^25^, and this mouse was also crossed onto the *TKO* line to generate *TKO;Carm1*^*KO*^ mice (Fig. 3g). Adenoviral-Cre intratracheal lavage was used to induce the conditional alleles recombination, and initiate cancer development. Both the loss of CARM1 and the mutation of the CARM1 methylation site on NFIB in the TKO mouse model resulted in dramatically reduced lung tumor burden, as well as the levels of the tumor cell proliferation biomarker, phospho-H3 (Fig. 3h–j). Both *TKO;Nfib*^*R388K*^ and *TKO;Carm1*^*KO*^ mutant mice displayed significantly prolonged survival over the *TKO* control (Fig. 3k). Both models also display a striking decrease in liver metastasis (Fig. 3l). Loss of NFIB methylation was confirmed in both mouse models by Western blotting with the NFIBme2a antibody (Fig. 3m). These results support a role for CARM1-mediated arginine methylation of NFIB in the development of SCLC.

### CARM1 and NFIB share the ability to promote an open chromatin state

**Fig. 4:**
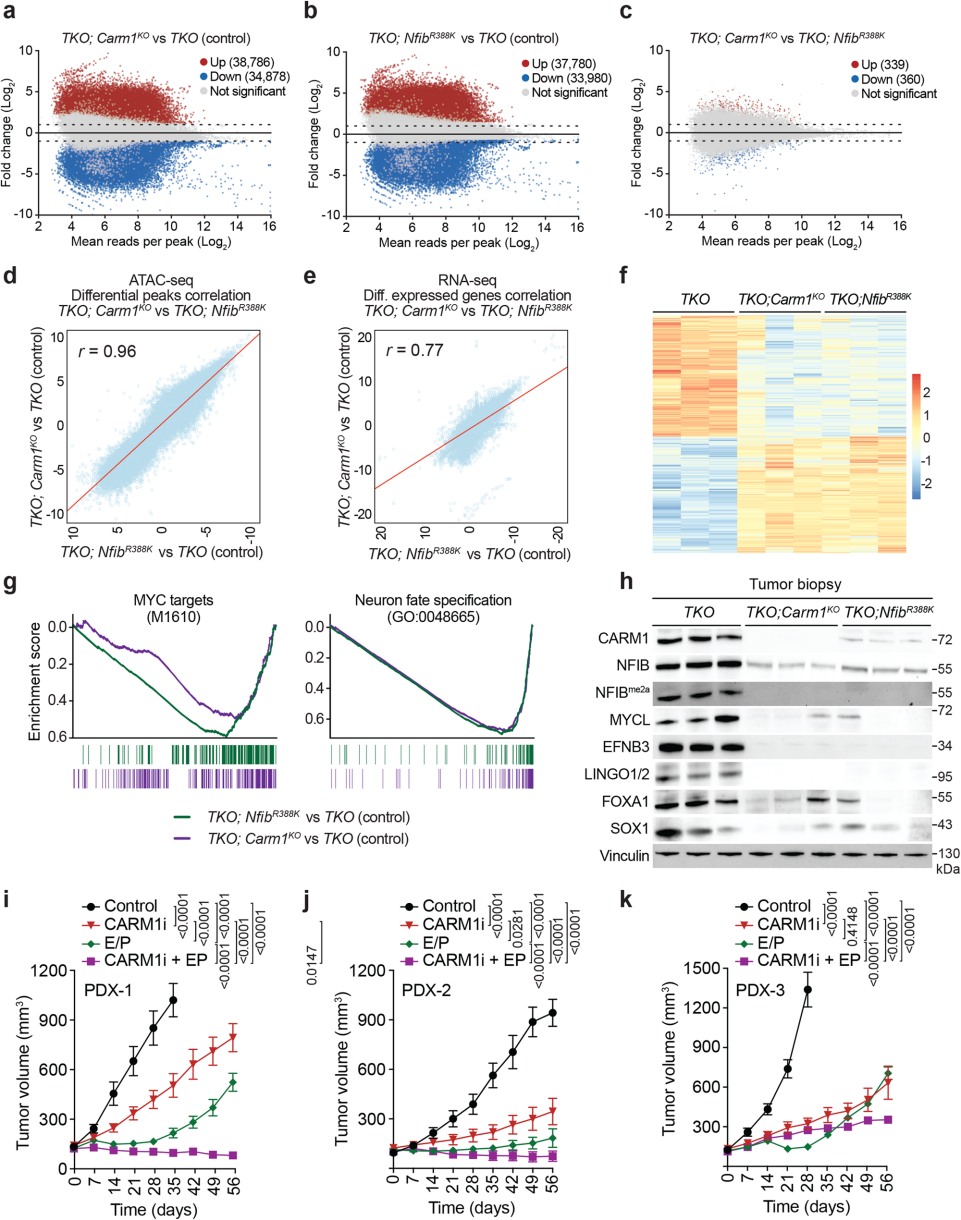
CARM1 methylation of NFIB regulates chromatin accessibility of SCLC cell to promote tumor growth. **a**–**c** ATAC-seq analysis of regions of differential chromatin accessibility in *TKO* (control) vs *TKO;Nfib*^*R388K*^ vs *TKO;Carm1*^*KO*^. **d** Correlation of differential chromatin accessibility between control, NFIB mutant and CARM1 knockout tumors collected from *n* = 3 independent mice for each group. Pearson’s correlation coefficient (*r*) was calculated. **e** Correlation of differential gene expression between control, NFIB mutant and CARM1 knockout tumors collected from n = 3 independent mice for each group. Pearson’s correlation coefficient (*r*) was calculated. **f** Gene clusters analysis of the expression changes detected by RNA-seq. **g** The CARM1-NFIB axis regulates multiple oncogenic programs. Examples of top gene set enrichment analysis (GSEA) signatures associated with CARM1 and NFIB K388 methylation deficiency in cancer cells isolated from *TKO* (control) vs. *TKO;Nfib*^*R388K*^ vs. *TKO;Carm1*^*KO*^ mutant mice. Normalized enrichment scores (NES) and false discovery rate (FDR) are provided (detailed statistics description in “Methods”). **h** Immunoblots with the indicated antibodies of tumor biopsy lysates from *TKO* (control), *TKO;Nfib*^*R388K*^, *TKO;Carm1*^*KO*^ mutant mice. Two independent samples are shown for each genotype. Vinculin shown as a loading control. **i**–**k** CARM1 inhibition attenuates the growth of three SCLC PDX models and in combination with Etoposide and Cisplatin (E/P) lead to tumor regression in NSG mice. Tumor volume quantification and immunoblots of the PDX were treated as indicated (*n* = 6 mice per group; *P* values were determined by two-way ANOVA with Tukey’s testing for multiple comparisons, data represent mean ± SEM).

To gain a molecular understanding of the NFIB/CARM1 pathway, we perform ATAC-seq and RNA-seq on the same tumor biopsies obtained for *TKO*, *TKO;Nfib*^*R388K*^ and *TKO;Carm1*^*KO*^ mutant mice. ATAC-seq revealed massive changes in chromatin accessibility. Mapping of the accessibility in different genomic regions reveals that *TKO;Nfib*^*R388K*^ and *TKO;Carm1*^*KO*^ show similar patterns compared to *TKO* (Supplementary Fig. 4a). A more detailed analysis of all changes in chromatin accessibility revealed that loss of CARM1 or mutation of NFIB cause over 30,000 sites of up- and downregulation (Fig. 4a, b). Importantly, when changes between *TKO;Nfib*^*R388K*^ and *TKO;Carm1*^*KO*^ are compared, there is almost no difference in accessibility (Fig. 4c), which implies that the chromatin changes observed in *TKO;Nfib*^*R388K*^ and *TKO;Carm1*^*KO*^ are matching. This is also true for the RNA-seq changes observed between tumor samples (Supplementary Fig. 4b). The similarity of the accessibility and expression changes, between *TKO;Nfib*^*R388K*^ and *TKO;Carm* ^*KO*^ tumors, is further validated by measurement of the linear correlation of these two data sets (Fig. 4d, e), the apparent grouping of *TKO;Nfib*^*R388K*^ and *TKO;Carm* ^*KO*^ when gene cluster analysis is performed on the transcriptome changes (Fig. 4f) and the principal component analyses (Supplementary Fig. 4c, d). As would be expected from these parallels, *TKO;Nfib*^*R388K*^ and *TKO;Carm* ^*KO*^ tumors display overlap in the Venn diagram depicting differentially expressed genes and chromatin accessibility changes (Supplementary Fig. 4e) and share GSEA signatures (Fig. 4g and Supplementary Fig. 5a). In addition, we found that previously published^27^ NFIB ChIP-seq peaks obtained from murine KP1 cells significantly overlapped with the down-regulated ATAC-seq peaks in *TKO;Nfib*^*R388K*^ and *TKO;Carm1*^*KO*^ versus *TKO* control of which over 93% shared peaks between *TKO;Nfib*^*R388K*^ and *TKO;Carm1*^*KO*^ (Supplementary Fig. 4f). As an example of some of the commonly regulated targets, which require both CARM1 and the NFIB methylation site, we show the browser tracks for LINGO1, SOX1, EPHB3, FOXA and MYCL (Supplementary Fig. 5b) and validate that these changes also occur at the protein level (Fig. 4h). Thus, CARM1 methylates NFIB and together they cooperate to reshape chromatin and coregulate gene expression.

### CARM1 small molecule inhibitors have therapeutic potential for the treatment of SCLC

Importantly, transcription factors like NFIB are considered “undruggable” targets^48^. However, as an enzyme, CARM1 is the therapeutically tractable target, and several potent and specific CARM1 inhibitors (CARM1i) have recently become available^31–33^; these inhibitors are active in in vivo mouse models. We thus tested the hypothesis that CARM1i may be of therapeutic value for SCLC by comparing it to the standard of care, which is etoposide and cisplatin (EP) chemotherapy. Three independent SCLC patient-derived xenograft (PDX) models were tested with EP, CARM1i, and EP + CARM1i. Monotherapy with CARM1i or EP could significantly attenuate tumor growth in all three models but was insufficient to lead to cancer regression (Fig. 4i–k). Strikingly, the EP + CARM1i combination led to the most dramatic effect on tumor volume and led to partial regression in two out of three PDXs. Thus, pre-clinical studies have provided four independent lines of evidence (complementation assays using xenografts; a *Nfib*^*R388K*^ knock-in mouse, a CARM1 conditional knockout mouse, and CARM1 inhibitors) supporting CARM1 as a potential therapeutic target for SCLC patients.

## Discussion

NFIB functions as an oncogene in small cell lung cancer^26^, by promoting SCLC metastasis and regulating chromatin accessibility^27,28,49,50^. There are also NFIB-independent mechanisms for SCLC metastases which involve the loss of the histone methyltransferase KMT2C^51^. Our study shows that loss of NFIB methylation inhibits the proliferation of SCLC cell lines as well as the progression of SCLC in mice (Fig. 3). These data further establish that the methylation of NFIB by CARM1 promotes the development of SCLC. Besides SCLC, NFIB was also reported to be amplified in ER-negative breast cancer and esophageal squamous cell carcinoma^52–54^, with possible oncogenic roles in these cancers. Furthermore, NFIB is reported to interact with transcription regulators to modulate its downstream transcription events. It was first shown to bind adjacent to the glucocorticoid receptor (GR) docking sequence on the promoter region of WAP gene and synergistically regulate the transcription of WAP^55^. Subsequent studies confirmed the association between NFIB and GR in gene regulation^56,57^. An interaction between NFIB and RFX1 was reported, and these transcription factors work together to modulate the expression of human growth hormone^58^. NFIB can regulate the transcription of androgen-dependent genes as well as estrogen responsive genes, by interacting with FOXA1^59,60^. Interestingly, tumor samples from *TKO;Nfib*^*R388K*^ and *TKO;Carm1*^*KO*^ mice display reduced FOXA1 expression (Fig. 4h and Supplementary Fig. 4e), suggesting the existence of a possible feedback loop. Moreover, NFIB can interact with FOXP2 to activate neuronal maturation genes^61^, and with KDM4D to regulate the expression of adipogenic regulators^62^. In this study, we report the arginine methylation-dependent interaction between NFIB and TRIM29 (Fig. 2), adding to our mechanistic understanding of how NFIB transcription programs are regulated.

A functional link between NFIB and TRIM29 has been made before. Indeed, a comparison of transcription regulatory networks in breast cancer, identified an overlapping TRIM29/NFIB risk-associated regulon^63^. Within a network, regulons overlap because different transcription factors and co-factors can regulate similar gene sets, which suggests that components of overlapping regulons work together. TRIM29 can regulate other transcriptional networks apart from NFIB. Our data shows that TRIM29 is a positive regulator of the transcription of NFIB target genes. This coactivator function of TRIM29 has been reported for MMP9 and p63, in non-small cell lung cancer and cervical cancer cells, respectively^64,65^. How TRIM29 functions as a coactivator is not clear. However, the ability of TRIM29 to interact with DNA repair proteins may provide mechanistic hint. Indeed, DNA-PK, XRCC6 and RUVBL1/2 were identified as highly ranked TRIM29 binders by both us and others^42^ (Fig. 2j). DNA-PK has numerous functions in the regulation of transcription, including (1) the phosphorylation of the general transcription factors TBP and TFIIB that stimulates basal transcription^66^; (2) the regulation of transcriptionally poised RNAPII^67^; (3) its presence at active sites of transcription^68^; and (4) its ability to co-regulator the androgen receptor^69^. The DNA-PK Ku regulatory proteins (XRCC6/5) were also identified as TRIM29 binders, and early studies using extracts from either DNA-PK- or XRCC6/5-null cells displayed decreased transcription of multiple promoters^70^. Moreover, RUVBL1/2, also known as Pontin and Reptin, have numerous roles in the control of transcription^71^. We also found that TRIM29 binds PRMT1 and the SWI/SNF complex (Fig. 2j), which both have clear roles in transcriptional regulation.

**Fig. 5:**
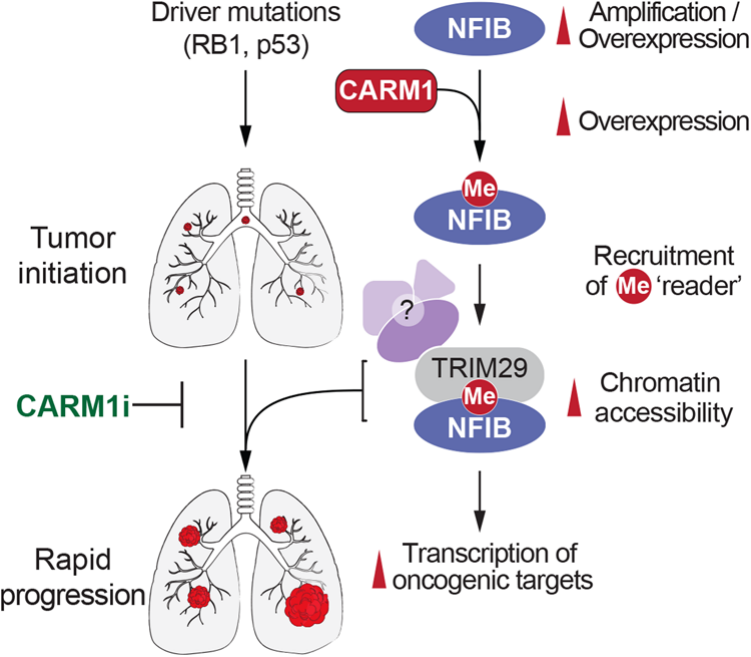
A model of CARM1-mediated NFIB regulation and SCLC therapeutic intervention. NFIB is methylated by CARM1, which subsequently recruits TRIM29 and possibly other effectors proteins. TRIM29 itself likely part of a protein complex that promotes the transcriptional activity of NFIB. CARM1 inhibitors (CARM1i) will attenuate the oncogenic properties of NFIB amplification.

SCLC accounts for approximately 15% of lung cancer and is a highly malignant and nearly uniformly fatal disease. To date, no targeted therapy has been approved for SCLC and the disease remains commonly treated with conventional chemotherapy, inevitably leading to acquired resistance and relapse. Lack of novel therapeutics is in part due to the unique biology of SCLC, which is driven by mutations in tumor suppressors and amplifications of transcription factors like NFIB and MYC. Indeed, dysregulated transcriptional programs can drive transformation, giving rise to what has been termed “transcriptional addiction” in cancer^72^. Transcription factors are difficult proteins to target with small molecule inhibitors because of their lack of enzymatic activity for chemical intervention. However, transcription factors can be modified by enzymes to regulate their activity, and they can recruit proteins with enzymatic activity to function as transcriptional coregulators. These enzymes could serve as therapeutic targets as a workaround for this issue. Currently, the therapeutic standard of care for SCLC is etoposide and cisplatin (EP) chemotherapy. We have shown that arginine methylation of NFIB is important for the progression of small cell lung cancer (Figs. 3 and 4), and elimination of this methylation either by removal of CARM1 or by mutation of the NFIB methylation site, significantly prolongs the survival of the SCLC mouse model (Fig. 3). Unlike NFIB, CARM1 activity can be efficiently inhibited by a number of small molecule compounds. Some of these compounds have exhibited anti-tumor activity in preclinical studies of leukemia and lymphoma^34,35^. In the future, the treatment of an SCLC GEMM with CARM1 inhibitors will further determine their therapeutic values of targeting this pathway for this cancer type (Fig. 5).

## Methods

### Plasmids

Stable cells were generated using pLenti-CMV and CRISPR/Cas9 knockouts using pLentiCRISPRv2 lentiviral vectors. Mouse *Nfib* or *Nfic* were cloned in between BglII and SalI restriction sites of pEGFP-C1 vector to generate GFP-NFIB and GFP-NFIC constructs, respectively. Flag-tagged TRIM29, HAT1, RBM14, SMARCD2 and GST-tagged TRIM29 and TUBB4B were purchased from Biomatik. The truncated forms of TRIM29 were generated by standard cloning strategies using primers listed the Supplementary Table 1. TDRD3 expression plasmids were described previously^19^.

### Virus production, infection, and selection

Lentivirus were generated using psPAX2 and VSV-G packaging plasmids. For virus production, 5 × 10^6^ HEK293T cells were plated into 10 cm dishes and transfected with the plasmids of interest using PEI and appropriate packaging plasmids. Medium was changed 6 h later. Supernatants were collected at 48 h, passed through 0.2 μm filters and applied at 50% concentration to target cells. Two days after infection, cells were subjected to selected with Puromycin (2 μg/mL) or Blastcidin (5 μg/mL).

### Generation of NFIBK388me2a-specific antibody

The NFIB methyl-specific antibody (NFIB^K388me2a^) was raised using peptide corresponding to human NFIB Ac-SHPTI(R-uMe2)YPPHC-amide, which contains R388me2a residue. The peptide was used as antigen to immunize rabbits. Immunization and antiserum production were performed by Covance Immunology Services. Rabbit immunoglobulins were affinity purified against NFIB^R388me2a^ peptide and negatively selected against the non-methylated NFIB^R388me0^ peptide from mixed sera.

### Immunoblot analysis and immunoprecipitation

For western blot analysis, cells were lysed in RIPA buffer with 1 mM PMSF and protease inhibitor cocktail. Protein concentration was determined using the Coomassie plus assay. Protein samples were resolved by SDS-PAGE and transferred to a PVDF membrane. The following antibodies were used (at the indicated dilutions): NFIB (Bethyl #A303-566A, 1:2000), NFIB^R388me2a^ (made in house, 1:5000), CARM1 (Bethyl #A300-421A, 1:2000), FLAG (Sigma-Aldrich #F1804, 1:2000), Vinculin (Cell Signaling Technology #13901, 1:2000), TRIM29 (Santa Cruz #sc166718, 1:1000), pan-ADMA (made in house, 1:2000), pan-SDMA (made in house, 1:2000), GST (made in house, 1:1000), TDRD3 (Millipore #MABE1042, 1:1000), H3R17me2a (Millipore #07-214, 1:1000) and Actin (Sigma-Aldrich # A1978, 1:10,000). Secondary antibodies were used at 1:5000 or 1:10000 dilution. Protein bands were visualized using ECL detection reagent. For immunoprecipitation, cells were harvested in PBS buffer then pelleted by centrifugation and resuspended in lysis buffer (50 mM Tris pH 8, 150 mM NaCl, 1 mM EDTA, 0.5% NP40, 10% glycerol, supplemented with protease inhibitor cocktail). Lysates were incubated with manufacturer recommended amount of antibody for 2 h at 4 °C with rotation then with protein A/G Magnetic beads for 1 h at 4 °C and washed three times. Proteins were eluted with Laemmli buffer and analyzed by immunoblotting. For immunoprecipitation with the mouse tissue, 2–3-month-old mice were sacrificed, then the lungs and spleens were homogenized and lysed in the same lysis buffer. 2 µg of the Bethyl NFIB antibody was used for the immunoprecipitation as described above.

### Peptide pulldown

Full length and truncated GST-TRIM29 were expressed and purified from *E. coli*. Unmethylated or methylated NFIB peptides and indicated GST proteins were incubated in NP-40 buffer (50 mM pH 7.5 Tris-HCl, 150 mM NaCl, 0.1% NP-40, 5 mM EDTA, 5 mM EGTA, 1.5 mM MgCl_2_) supplemented with the protease inhibitor cocktail, and shaken for 1 h at 4 °C. The mixtures were then incubated with Streptavidin T1 Dynabeads for 45 min at 4 °C. The eluted samples were loaded on SDS-PAGE gel and detected by Western blots using anti-GST antibody.

### Mass spectrometry

Immunoprecipitated proteins from MEFs or HeLa cells were loaded into SDS-PAGE gel and electrophorized for 20 min. The gel plug containing the proteins of interest was excised and subjected to in-gel digestion with trypsin. Peptides were eluted and analyzed by nanoflow LC-MS/MS using the Thermo Ultimate 3000 RSLCnano UPLC in line with the Orbitrap Fusion hybrid mass spectrometer. The resulting spectra were assigned to peptide sequences using Proteome Discoverer software (Thermo) with the Sequest-HT database search algorithm. The appropriate Uniprot (mouse or human) database was used along with a list of common protein contaminants. Scaffold software (Proteome Software) provided data validation. The protein identifications were filtered for minimum 2 peptide at 95% confidence and 99% protein confidence. Lists of credible methyl-peptide sequence assignments were generated for samples from MEFs and lists of credible peptide sequence assignments were generated for samples from HeLa and H69 cells.

#### Mass spectrometry-based proteomic analysis to identify methylation sites

pan-ADMA antibodies were incubated with cell lysates from Carm1 WT MEFs or KO MEFs, respectively. Methylated proteins bound to the antibodies were enriched with protein A/G magnetic beads. The magnetic beads were then washed and boiled in Laemmli buffer. Eluted proteins were loaded to SDS-PAGE gel for the following mass spectrometry analysis.

#### Mass spectrometry analysis of methyl-sensitive binders

HeLa cells were transiently transfected with GFP-NFIB-WT or GFP-NFIB-R388K plasmids. 24 h post transfection, cells were harvested and lysed in NP-40 buffer. Proteins binding to GFP-fused NFIB WT or mutant were enriched using the GFP-trap kit, following the manufacturer’s instruction. Eluted proteins were loaded to SDS-PAGE gel for the following mass spectrometry analysis. Protein lists were filtered to remove those with peptide counts from the control R388K sample.

#### Mass spectrometry analysis of TRIM29 binding proteins

H69 cells stably expressing GFP or GFP-TRIM29 were harvested and lysed in NP-40 buffer. Proteins binding to GFP or GFP-TRI29 were enriched using the GFP-trap kit, following the manufacturer’s instruction. Eluted proteins were loaded to SDS-PAGE gel for the following mass spectrometry analysis. Protein lists were filtered to remove those with peptide counts from the control GFP sample.

### Luciferase assays

293T cells were co-transfected with GFAP-luc reporter together with indicated expression plasmids. Twenty-four hours post transfection, cells were applied for the detection of luciferase activity per the manufacturer’s instructions. Briefly, cells were lysed with passive lysis buffer at room temperature for 15 min. Luciferase reagents and Stop & Glo reagents were subsequently added to the lysates to obtain the readings for firefly and *Renilla* luciferase activities.

### Chromatin Immunoprecipitation (ChIP)

In brief, HeLa cells were crosslinked and lysed with ChIP lysis buffer (50 mM pH8.0 Tris-HCl, 140 mM NaCl, 1 mM EDTA, 10% glycerol, 0.5% NP-40, 0.25% Triton X-100, supplemented with protease inhibitor cocktail), followed by sonication to shear the DNA to 200–1000 bp. 2 µg of the Bethyl NFIB antibody was incubated with the DNA at 4 °C overnight. Protein A/G magnetic beads were then added to the antibody/DNA mix and incubated at 4 °C for 1 h. Beads were washed, and the DNA was eluted, reversed crosslinked and purified using a DNA purification kit. RT-qPCR analyses were performed on immunoprecipitated DNA using specific primers (Supplementary Table 1).

### Quantitative RT-PCR

For quantitative RT-PCR, RNA was extracted using TRIzol Reagent according to the manufacturer’s instructions, cDNA synthesis was obtained using the Superscript First-strand Synthesis kit. Quantitative real-time PCR analysis was performed on ABI 7900HT using SYBR Green Master Mix following the manufacturer’s manual and specific primers (Supplementary Table 1). The expression of each gene was normalized to GAPDH. Relative expression levels were calculated using the ΔΔCT method.

### Genomic, Epigenomic and transcriptome analysis

#### ChIP-sequencing and data analysis

Three independent antibodies were used in the ChIP-seq analysis for the global distribution of NFIB in HeLa cells. Two NFIB antibodies from Abcam and Bethyl were used for regular HeLa cells, while GFP antibody was used for HeLa cells with stable GFP-NFIB overexpression. Two technical replicates of DNA libraries each antibody and the corresponding total input libraries were prepared using NEB Ultra DNA library prep kit and subjected to sequencing on Illumina HiSeq 3000 systems. Sequenced DNA reads were mapped to the human genome hg19 using Illumina analysis pipeline CASAVA (version 1.8.2) and only the reads mapping to a unique position were retained. 26–33 million reads were generated per sample. 81–93% of the total reads were mapped to the human genome, while 68–78% of the total reads were uniquely mapped. To avoid PCR bias, only one copy for multiple reads that mapped to the same genomic position was retained for further analysis. In the end, 17–23 million reads were used in peak calling and downstream analyses. To obtain the NFIB peaks pulled down by each antibody, the two replicate samples were merged to a merged sample and peak calling was performed on both the merged sample and each individual replicate by MACS using total input DNA as the negative control. The window size was set as 300 bp and the *P* value cutoff was set as 1 × 10^−5^. The peaks overlapped with ENCODE blacklisted regions were removed. Then the peaks by the three antibodies were merged (allowing at least 1 bp overlap) and the merged peaks that overlapped with the peaks by all the three antibodies were deemed as the high confident loci directly bound by NFIB (i.e., NFIB^YYY^). Each peak in NFIB^YYY^ was assigned to the gene that has the closest transcription start site (TSS) to it. And the peak was classified according to its location to the gene: upstream (−50 k to −5 k from TSS), promoter (−5 k to +0.5 k from TSS), exon, intron, the transcription end site (TES) (−0.5 k to +5 k from TES), and downstream (+5 k to +50 k from TES). The genes used to annotate the peaks is RefSeq genes downloaded from UCSC Genome Browser on July 17, 2015. For signal track, each read was extended by 150 bp to its 3’ end. The number of reads on each genomic position was rescaled to normalize the total number of reads to 10 M and averaged over every 10 bp window. The normalized values were displayed in UCSC genome browser. The signal tracks of merged samples are shown. The H3R17me2a antibody was used for the global distribution of CARM1 substrates in HeLa cells. The analysis for H3R17me2a, including mapping and peak calling, was the same as in^9^ (briefly, the same as NFIB, except the mapping was done using bowtie (version 0.12.8) and the *P* value cutoff for peak calling was 1 × 10^−6^ to control the empirical FDR below 0.05). 46 and 52 million reads were generated for H3R17me2a and total input, respectively. 95 and 97% of the total reads were mapped to the human genome, with 73 and 74% uniquely mapped. In the end, 29 (H3R17me2a) and 38 (total input) million reads were used in the peak calling for H3R17me2a.

#### RNA-sequencing and gene set enrichment analysis

Total RNA was extracted from FACS sorted cancer cell using tdTomato reporter from *TKO*; *tdTomato, TKO;Carm1*^*KO*^; *tdTomato*, and *TKO;Nfib*; *tdTomato* mutant mice lung tumors using Trizol reagent. RNA-seq libraries were constructed and sequenced by Novogene. The RNA-seq libraries were sequenced using Illumina platform (pair end 150 bp analysis). Low-quality and adapter-containing reads were trimmed using trim-galore package under paired-end mode, any reads shorter than 50 bp were removed. The remaining trimmed sequences were mapped to the reference genome (mm10) with hisat2 under default settings. We used htseq-count to count the mapped reads number on every mm10 Refseq transcript. Differential gene expression analysis was performed with the DESeq2 package. Genes with FDR < 0.1 and Log_2_ fold change ≥0.8 were defined as upregulated genes, and genes with FDR < 0.1 and Log_2_ fold change ≤−0.8 were defined as down-regulated genes. The read count for all genes was calculated using featureCounts and then normalized by DESeq2 to obtain normalized counts. The normalized count matrix was served as input for gene set enrichment analysis (GSEA) using previously described software and the Molecular Signatures Database (MSigDB).

#### ATAC-sequencing and data analysis

Cancer cell lines were FACS sorted cancer cell using tdTomato reporter from *TKO*; *tdTomato, TKO;Carm1*^*KO*^; *tdTomato*, and *TKO;Nfib*; *tdTomato* mutant mice lung tumors. For each genotype 50,000 cells were collected, washed in phosphate-buffered saline, and incubated in 50 μL of lysis buffer (10 mM Tris HCl, 10 mM NaCl, 3 mM MgCl2, 0.1% NP40, pH 7.4) for 5 min on ice followed by centrifugation (500 × *g* at 4 °C for 10 min) and removal of supernatant. Transposition and library preparation were performed using the Nextera workflow. Cells were resuspended in 50 μL transposition reaction mix (2.5 μL TDE1 Tn5 transposase and 1 μL 0.5% digitonin in 1× Tagment DNA buffer) and incubated at 37 °C for 30 min. The reaction was quenched using 1 μL 10% sodium dodecyl sulfate and bead purification was performed using Agencourt AmpureXP beads (Beckman Coulter; A63881) according to the manufacturer’s instructions. Tagmented DNA was amplified using i7 and i5 indexing adapters using a five-cycle PCR program according to the manufacturer’s instructions, size-selected using AmpureXP beads to remove species >600 bp, and then normalized and pooled for sequencing. ATAC-seq libraries were sequenced using the Illumina NovaSeq PE150, 90G. Next, adaptor sequence trimming, mapping to the mouse (mm10) reference genome using Bowtie2 and PCR duplicate removal using Picard Tools were performed. Peaks were called using MACS2 and filtered to remove putative copy number altered regions. The number of reads/peak was determined for each sample using bedtools multicov, and the relative sequencing depth was estimated using a set of “housekeeping” peaks at transcription start sites of genes that were uniformly expressed across all tumor genotypes. Differential accessibility was assessed using DESeq2. Unless otherwise stated, regions were called differentially accessible if the absolute value of the log_2_ fold change was 0.5 at an FDR < 0.1.

### Histology and immunohistochemistry

fixed in 4% buffered formalin for 24 h and stored in 70% ethanol until paraffin embedding. 3 μm sections were stained with hematoxylin and eosin (HE) or used for immunohistochemical studies. Immunohistochemistry (IHC) was performed on formalin-fixed, paraffin embedded mouse and human tissue sections using a biotin-avidin conjugated HRP (Vectastain ABC kit). The following antibodies were used (at the indicated dilutions): CARM1 (1:1000) and NFIB (1:1000). Sections were developed with DAB substrate and counterstained with hematoxylin. Pictures were taken using a PreciPoint M8 microscope equipped with the PointView software. Analysis of the tumor area and IHC analysis was done using ImageJ software.

### Quantification and statistical analysis

Please refer to the Figure Legends for description of sample size (*n*) and statistical details. All values for n are for individual mice or individual sample. Sample sizes were chosen based on previous experience with given experiments. Cell culture assays have been performed in biological triplicates. Differences were analyzed by log-rank, two-tailed unpaired Student’s t test, two-way ANOVA with Tukey’s testing for multiple comparisons using Prism 8 (GraphPad) or Excel.

### Animal models

*Trp53*^*loxP/loxP*^*, Rb1*^*LoxP/LoxP*^, *Rbl1*^*LoxP/LoxP*^, *Carm1*^*LoxP/LoxP*^, *Rosa26*^*LSL-tdTomato*^ mice have been described before^25,47^. *Nfib*^*R388K*^ knockin mice were generated using sgRNA targeting the arginine 388 residue of NFIB, donor DNA containing the R-to-K mutation were designed by Horizon Discovery. sgRNA, donor DNA, as well as Cas9 protein were micro-injected into 1-cell embryos. The sequences of the sgRNA and donor DNA are in the oligo list. The injected embryos were then transferred into pseudopregnant recipient female mice. Founder mice were screened for the R388K mutation in NFIB. Five of the 14 pups born were identified with mutant KI allele; three founders were further characterized. The founders were backcrossed with C57BL/6 strain background mice for three generations to separate any off-target events from the KI allele. Mice were maintained on a mixed C57BL/6;FVB129/N strain background and we systematically used littermates as controls in all the experiments. Immunocompromised female NSG mice (*NOD.SCID-IL2Rg-/-*) mice were utilized for transplantation studies. All experiments were performed on 6 to 10-week-old female animals. All animals were numbered and experiments were conducted in a blinded fashion. After data collection, genotypes were revealed and animals assigned to groups for analysis. For treatment experiments, mice were randomized. None of the mice with the appropriate genotype were excluded from this study or used in any other experiments. All mice were co-housed with littermates (2–5 per cage) in pathogen-free facility with standard controlled temperature of 72 °F, with a humidity of 30–70%, and a light cycle of 12 h on/12 h off set from 7 am to 7 pm and with unrestricted access to standard food and water under the supervision of veterinarians, in an AALAC-accredited animal facility at the University of Texas M.D. Anderson Cancer Center (MDACC). Mouse handling and care followed the NIH Guide for Care and Use of Laboratory Animals. All animal procedures followed the guidelines of and were approved by the MDACC Institutional Animal Care and Use Committee (IACUC protocol 00001636, PI: Mazur). Tumor size was measured using a digital caliper and tumor volume was calculated using the formula: Volume = (width)^2^ × length/2 where *length* represents the largest tumor diameter and *width* represents the perpendicular tumor diameter. The endpoint was defined as the time at which a progressively growing tumor reached 20 mm in its longest dimension as approved by the MDACC IACUC protocol (00001636, PI: Mazur) and in no experiments was this limit exceeded. Mice were euthanized with carbon dioxide.

### Administration of Cre for the small cell lung cancer mouse models

To generate tumors in the lungs of *Rb1*^*LoxP/LoxP*^, *Rbl2*^*LoxP/LoxP*^; *Tp53*^*LoxP/LoxP*^ (*TKO*); *Rb1*^*LoxP/LoxP*^, *Rbl2*^*LoxP/LoxP*^; *Tp53*^*LoxP/LoxP*^; *Carm1*^*LoxP/LoxP*^ (*TKO;Carm1*^*KO*^); *Rb1*^*LoxP/LoxP*^, *Rbl2*^*LoxP/LoxP*^; *Tp53*^*LoxP/LoxP*^; *Nfib*^*R388K*^ (*TKO;Nfib*^*R388K*^) and in combination with *Rosa26*^*LSL-tdTomato*^ (*dtTomato*) reporter mice, we used replication-deficient adenoviruses expressing Cre-recombinase (Ad-Cre) as previously described^47^. Briefly, 8-week-old mice were anesthetized by continuous gaseous infusion of 2% isoflurane for at least 10 min using a veterinary anesthesia system (D19 Vaporizer, Vetland Medical). Ad-Cre was delivered to the lungs by intratracheal installation. Prior to administration, Ad-Cre was precipitated with calcium phosphate to improve the delivery of Cre by increasing the efficiency of viral infection of the lung epithelium. Mice were treated with one dose of 5 × 10^6^ PFU of Ad-Cre (Baylor College of Medicine, Viral Vector Production Core). Mice were analyzed for tumor formation and progression at indicated times after infection. Tumor biopsies were collected and protein lysates prepared to confirm mutation of conditional alleles by immunoblotting.

### Cell lines cultures

293 T (ATCC #CRL-3216), H69 (ATCC #HTB-119), HeLa (ATCC #CCL2), H446 (ATCC #HTB-171), H209 (ATCC #HTB-172), CORL279 (Sigma-Aldrich #96020724), H2171 (ATCC #CRL-5929), SCLC21H (Accegen #ABC-TC605S), SW1271 (ATCC #CRL-2177), H526 (CRL-5811) and CORL47 (Sigma-Aldrich #92031915) cells were grown in Dulbecco’s modified Eagle’s medium (DMEM) supplemented with 10% fetal calf serum, 2 mM L-glutamine-penicillin–streptomycin. Cell lines were freshly purchased for this study. Primary mouse cancer cell lines were prepared from tumor biopsies isolated from the indicated mouse models. Primary mouse cancer cell lines were cultured in DMEM supplemented with 10% fetal calf serum, 2 mM L-glutamine–penicillin–streptomycin. All cells were cultured at 37 °C in a humidified incubator with 5% CO_2_. Cell lines were authenticated by short tandem repeat profiling and tested negative for mycoplasma. Cell growth was assessed using Alamar Blue assay and soft agar colony formation assay using standard methods. All cell lines in culture are routinely tested for mycoplasma contamination and their identity confirmed through the “Cytogenetics and Cell Authentication Core“, at MD Anderson Cancer Center if needed.

### Patient-derived cancer xenografts

Patient-derived xenografts (PDXs) were obtained from the NCI Patient-Derived Models Repository (PDMR), NCI-Frederick, Frederick National Laboratory for Cancer Research (Specimen ID: 638129-119-R, 541946-237-B). Briefly, surgically resected tumor specimens were obtained from deidentified patients with histologically confirmed SCLC. PDX#1 (638129-119-R) was derived from a chemotherapy naïve patient. PDX#2 (541946-237-B) was derived from a patient that received Carboplatin and Etoposide therapy for 3 months with partial response followed by disease progression. All tumor specimens were collected after written patient consent and in accordance with the institutional review board-approved protocols of the University of Texas M.D. Anderson Cancer Center (PA19-0435, PI: Mazur). Patient-derived xenograft tumors were generated and propagated by transplanting small tumor fragments isolated directly from surgical specimens subcutaneously into NSG mice. Whole Exome Sequencing was performed and cancer gene panel analysis revealed that PDXs are carrying characteristic for SCLC mutations, specifically PDX#1: *RB1*^*p.X473_splice*^*; TP53*^*pX224_splice*^*; CREBBP*^p.E371Rfs*56^*; MSH3*^p.A61Pfs*25^ and PDX#2: *RB1*^*p.X738_splice*^*; TP53*^*pR249G*^*; KMT2D*^p.A1390Qfs*27^*; MSH3*^p.V1192Cfs*2^. When tumors became palpable, they were calipered to monitor growth kinetics. For therapy studies mice were treated as indicated with Cisplatin, Etoposide in vehicle 0.9% saline and TP064 CARM1 inhibitor in vehicle 10% (2-hydroxypropyl)-β-cyclodextrin. Control and monotherapy animals underwent the same procedure but received vehicle treatment. Tumor volume was calculated using the formula: Volume = (width)2 × length/2 where *length* represents the largest tumor diameter and *width* represents the perpendicular tumor diameter.

### Cancer cell-derived mouse xenograft

For xenograft studies, human SCLC cell line H446 and H69 were transduced with lentivirus expressing sgRNA/Cas9 targeting CARM1 or NFIB and selected with hygromycin. Next, the cells were transduced with lentivirus expressing wildtype or mutant forms of CARM1 and NFIB as indicated and selected with puromycin. The cells were trypsinized and singularized. The trypsin was washed with excess growth medium and the cells were counted. The cells were then resuspended in PBS and mixed with matrigel (1:1) at a density of 2 × 10^7^ cells per ml and kept on ice until injection. Next, 100 μl of the cell suspension was injected subcutaneously into the hind flanks of NSG mice. When tumors became palpable, they were calipered to monitor growth kinetics. Tumor volume was calculated using the formula: Volume = (width)2 × length/2 where *length* represents the largest tumor diameter and *width* represents the perpendicular tumor diameter. For therapy studies, mice were treated as indicated. Control animals received vehicle treatment.

### Reporting summary

Further information on research design is available in the Nature Portfolio Reporting Summary linked to this article.

## Supplementary information

Supplementary Information

Description of Additional Supplementary Files

Supplementary Data 1

Supplementary Data 2

Supplementary Data 3

Supplementary Data 4

Reporting Summary

## Data Availability

The RNA-seq and ATAC-seq data generated in this study has been deposited in the GEO database under accession code GSE195843. The ChIP-seq data generated in this study has been deposited in the GEO database under accession number GSE219265. The mass spectrometry proteomics data have deposited to the ProteomeXchange via PRIDE with the identifier PXD038237 (http://proteomecentral.proteomexchange.org/cgi/GetDataset?ID=PXD038217), and via MASSive with the identifier MSV000090746 (https://massive.ucsd.edu/ProteoSAFe/result.jsp?task=af3cf7b04fa54f328a9be4d90dd2ae29&view=advanced_view#%7B%7D). All data supporting the findings of this study are available within the article and its supplementary information files. Source data are provided with this paper.

## Source data

Source Data

## References

[1] Yang Y, Bedford MT (2013). Protein arginine methyltransferases and cancer. Nat. Rev. Cancer.

[2] Guccione E, Richard S (2019). The regulation, functions and clinical relevance of arginine methylation. Nat. Rev. Mol. Cell Biol..

[3] Wu, Q., Schapira, M., Arrowsmith, C. H. & Barsyte-Lovejoy, D. Protein arginine methylation: from enigmatic functions to therapeutic targeting. *Nat. Rev. Drug Discov.*10.1038/s41573-021-00159-8 (2021). 10.1038/s41573-021-00159-8 33742187

[4] Blanc RS, Richard S (2017). Arginine methylation: the coming of age. Mol. Cell.

[5] Chen D (1999). Regulation of transcription by a protein methyltransferase. Science.

[6] Lee J, Bedford MT (2002). PABP1 identified as an arginine methyltransferase substrate using high-density protein arrays. EMBO Rep..

[7] Chevillard-Briet M, Trouche D, Vandel L (2002). Control of CBP co-activating activity by arginine methylation. EMBO J..

[8] Cheng D, Cote J, Shaaban S, Bedford MT (2007). The arginine methyltransferase CARM1 regulates the coupling of transcription and mRNA processing. Mol. Cell.

[9] Cheng D (2018). CARM1 methylates MED12 to regulate its RNA-binding ability. Life Sci. Alliance.

[10] Shishkova E (2017). Global mapping of CARM1 substrates defines enzyme specificity and substrate recognition. Nat. Commun..

[11] Ceschin DG (2011). Methylation specifies distinct estrogen-induced binding site repertoires of CBP to chromatin. Genes Dev..

[12] Wang L (2015). MED12 methylation by CARM1 sensitizes human breast cancer cells to chemotherapy drugs. Sci. Adv..

[13] Yu YS (2020). Pontin arginine methylation by CARM1 is crucial for epigenetic regulation of autophagy. Nat. Commun..

[14] Kawabe Y, Wang YX, McKinnell IW, Bedford MT, Rudnicki MA (2012). Carm1 regulates Pax7 transcriptional activity through MLL1/2 recruitment during asymmetric satellite stem cell divisions. Cell Stem Cell.

[15] Zhao HY, Zhang YJ, Dai H, Zhang Y, Shen YF (2011). CARM1 mediates modulation of Sox2. PLoS One.

[16] Vu LP (2013). PRMT4 blocks myeloid differentiation by assembling a methyl-RUNX1-dependent repressor complex. Cell Rep..

[17] Behera AK (2019). Functional interplay between YY1 and CARM1 promotes oral carcinogenesis. Oncotarget.

[18] Cote J, Richard S (2005). Tudor domains bind symmetrical dimethylated arginines. J. Biol. Chem..

[19] Yang Y (2010). TDRD3 is an effector molecule for arginine-methylated histone marks. Mol. Cell.

[20] Yang Y (2014). Arginine methylation facilitates the recruitment of TOP3B to chromatin to prevent R loop accumulation. Mol. Cell.

[21] Gayatri S, Bedford MT (2014). Readers of histone methylarginine marks. Biochim. Biophys. Acta.

[22] Harris L, Genovesi LA, Gronostajski RM, Wainwright BJ, Piper M (2015). Nuclear factor one transcription factors: Divergent functions in developmental versus adult stem cell populations. Dev. Dyn..

[23] Hsu YC (2011). Mesenchymal nuclear factor I B regulates cell proliferation and epithelial differentiation during lung maturation. Dev. Biol..

[24] O’Brien KB (2010). CARM1 is required for proper control of proliferation and differentiation of pulmonary epithelial cells. Development.

[25] Yadav N (2003). Specific protein methylation defects and gene expression perturbations in coactivator-associated arginine methyltransferase 1-deficient mice. Proc. Natl Acad. Sci. USA.

[26] Dooley AL (2011). Nuclear factor I/B is an oncogene in small cell lung cancer. Genes Dev..

[27] Denny SK (2016). Nfib promotes metastasis through a widespread increase in chromatin accessibility. Cell.

[28] Semenova EA (2016). Transcription factor NFIB is a driver of small cell lung cancer progression in mice and marks metastatic disease in patients. Cell Rep..

[29] Suresh S, Huard S, Dubois T (2021). CARM1/PRMT4: making its mark beyond is function as a transcriptional coactivator. Trends Cell Biol..

[30] Bao J (2019). Mouse models of overexpression reveal distinct oncogenic roles for different type I protein arginine methyltransferases. Cancer Res..

[31] Nakayama K (2018). TP-064, a potent and selective small molecule inhibitor of PRMT4 for multiple myeloma. Oncotarget.

[32] Drew AE (2017). Identification of a CARM1 inhibitor with potent in vitro and in vivo activity in preclinical models of multiple myeloma. Sci. Rep..

[33] Cai, X. C. et al. A chemical probe of CARM1 alters epigenetic plasticity against breast cancer cell invasion. *Elife*10.7554/eLife.47110 (2019). 10.7554/eLife.47110 PMC6917500 31657716

[34] Greenblatt SM (2018). CARM1 is essential for myeloid leukemogenesis but dispensable for normal hematopoiesis. Cancer Cell.

[35] Veazey KJ (2020). CARM1 inhibition reduces histone acetyltransferase activity causing synthetic lethality in CREBBP/EP300-mutated lymphomas. Leukemia.

[36] Guo A (2014). Immunoaffinity enrichment and mass spectrometry analysis of protein methylation. Mol. Cell Proteom..

[37] Grunder A (2002). Nuclear factor I-B (Nfib) deficient mice have severe lung hypoplasia. Mech. Dev..

[38] Steele-Perkins G (2005). The transcription factor gene Nfib is essential for both lung maturation and brain development. Mol. Cell Biol..

[39] Becker-Santos DD, Lonergan KM, Gronostajski RM, Lam WL (2017). Nuclear Factor I/B: A Master Regulator of Cell Differentiation with Paradoxical Roles in Cancer. EBioMedicine.

[40] Gronostajski RM (2000). Roles of the NFI/CTF gene family in transcription and development. Gene.

[41] Sims RJ (2011). The C-terminal domain of RNA polymerase II is modified by site-specific methylation. Science.

[42] Masuda Y (2015). TRIM29 regulates the assembly of DNA repair proteins into damaged chromatin. Nat. Commun..

[43] Zheng S (2013). Arginine methylation-dependent reader-writer interplay governs growth control by E2F-1. Mol. Cell.

[44] Shu T, Butz KG, Plachez C, Gronostajski RM, Richards LJ (2003). Abnormal development of forebrain midline glia and commissural projections in Nfia knock-out mice. J. Neurosci..

[45] Brun M (2009). Nuclear factor I regulates brain fatty acid-binding protein and glial fibrillary acidic protein gene expression in malignant glioma cell lines. J. Mol. Biol..

[46] Gobius I (2016). Astroglial-mediated remodeling of the interhemispheric midline is required for the formation of the Corpus Callosum. Cell Rep..

[47] Schaffer BE (2010). Loss of p130 accelerates tumor development in a mouse model for human small-cell lung carcinoma. Cancer Res..

[48] Yan C, Higgins PJ (2013). Drugging the undruggable: transcription therapy for cancer. Biochim. Biophys. Acta.

[49] Wu N (2016). NFIB overexpression cooperates with Rb/p53 deletion to promote small cell lung cancer. Oncotarget.

[50] Yang D (2018). Intertumoral heterogeneity in SCLC is influenced by the cell type of origin. Cancer Discov..

[51] Na F (2022). KMT2C deficiency promotes small cell lung cancer metastasis through DNMT3A-mediated epigenetic reprogramming. Nat. Cancer.

[52] Han W (2008). DNA copy number alterations and expression of relevant genes in triple-negative breast cancer. Genes Chromosomes Cancer.

[53] Moon HG (2011). NFIB is a potential target for estrogen receptor-negative breast cancers. Mol. Oncol..

[54] Yang ZQ (2001). A novel amplicon at 9p23 - 24 in squamous cell carcinoma of the esophagus that lies proximal to GASC1 and harbors NFIB. Jpn J. Cancer Res..

[55] Mukhopadhyay SS, Wyszomierski SL, Gronostajski RM, Rosen JM (2001). Differential interactions of specific nuclear factor I isoforms with the glucocorticoid receptor and STAT5 in the cooperative regulation of WAP gene transcription. Mol. Cell Biol..

[56] Lajoie M, Hsu YC, Gronostajski RM, Bailey TL (2014). An overlapping set of genes is regulated by both NFIB and the glucocorticoid receptor during lung maturation. BMC Genomics.

[57] Willi M, Yoo KH, Wang C, Trajanoski Z, Hennighausen L (2016). Differential cytokine sensitivities of STAT5-dependent enhancers rely on Stat5 autoregulation. Nucleic Acids Res..

[58] Norquay LD (2003). RFX1 and NF-1 associate with P sequences of the human growth hormone locus in pituitary chromatin. Mol. Endocrinol..

[59] Grabowska MM (2014). NFI transcription factors interact with FOXA1 to regulate prostate-specific gene expression. Mol. Endocrinol..

[60] Nanda JS (2020). Increased nuclear factor I/B expression in prostate cancer correlates with AR expression. Prostate.

[61] Hickey SL, Berto S, Konopka G (2019). Chromatin decondensation by FOXP2 promotes human neuron maturation and expression of neurodevelopmental disease Genes. Cell Rep..

[62] Choi JH, Lee H (2020). Histone demethylase KDM4D cooperates with NFIB and MLL1 complex to regulate adipogenic differentiation of C3H10T1/2 mesenchymal stem cells. Sci. Rep..

[63] Castro MA (2016). Regulators of genetic risk of breast cancer identified by integrative network analysis. Nat. Genet..

[64] Tang ZP, Cui QZ, Dong QZ, Xu K, Wang EH (2013). Ataxia-telangiectasia group D complementing gene (ATDC) upregulates matrix metalloproteinase 9 (MMP-9) to promote lung cancer cell invasion by activating ERK and JNK pathways. Tumour Biol..

[65] Masuda Y, Takahashi H, Hatakeyama S (2015). TRIM29 regulates the p63-mediated pathway in cervical cancer cells. Biochim. Biophys. Acta.

[66] Chibazakura T (1997). Phosphorylation of human general transcription factors TATA-binding protein and transcription factor IIB by DNA-dependent protein kinase–synergistic stimulation of RNA polymerase II basal transcription in vitro. Eur. J. Biochem..

[67] Dvir A, Stein LY, Calore BL, Dynan WS (1993). Purification and characterization of a template-associated protein kinase that phosphorylates RNA polymerase II. J. Biol. Chem..

[68] Ju BG (2006). A topoisomerase IIbeta-mediated dsDNA break required for regulated transcription. Science.

[69] Goodwin JF (2015). DNA-PKcs-mediated transcriptional regulation drives prostate cancer progression and metastasis. Cancer Cell.

[70] Woodard RL, Anderson MG, Dynan WS (1999). Nuclear extracts lacking DNA-dependent protein kinase are deficient in multiple round transcription. J. Biol. Chem..

[71] Gallant P (2007). Control of transcription by Pontin and Reptin. Trends Cell Biol..

[72] Bradner JE, Hnisz D, Young RA (2017). Transcriptional addiction in cancer. Cell.

