# Supplementary material for: The NFIB/CARM1 partnership is a driver in preclinical models of small cell lung cancer
Source: Nat Commun. 2023 Jan 23;14:363. doi: 10.1038/s41467-023-35864-y (PMC9870865; doi:10.1038/s41467-023-35864-y)
Supplement: Supplementary file 1 — Supplementary Information [file 41467_2023_35864_MOESM1_ESM.pdf]

The NFIB/CARM1 Partnership is a Driver in Preclinical Models of Small Cell Lung Cancer

Supplementary Figure 1

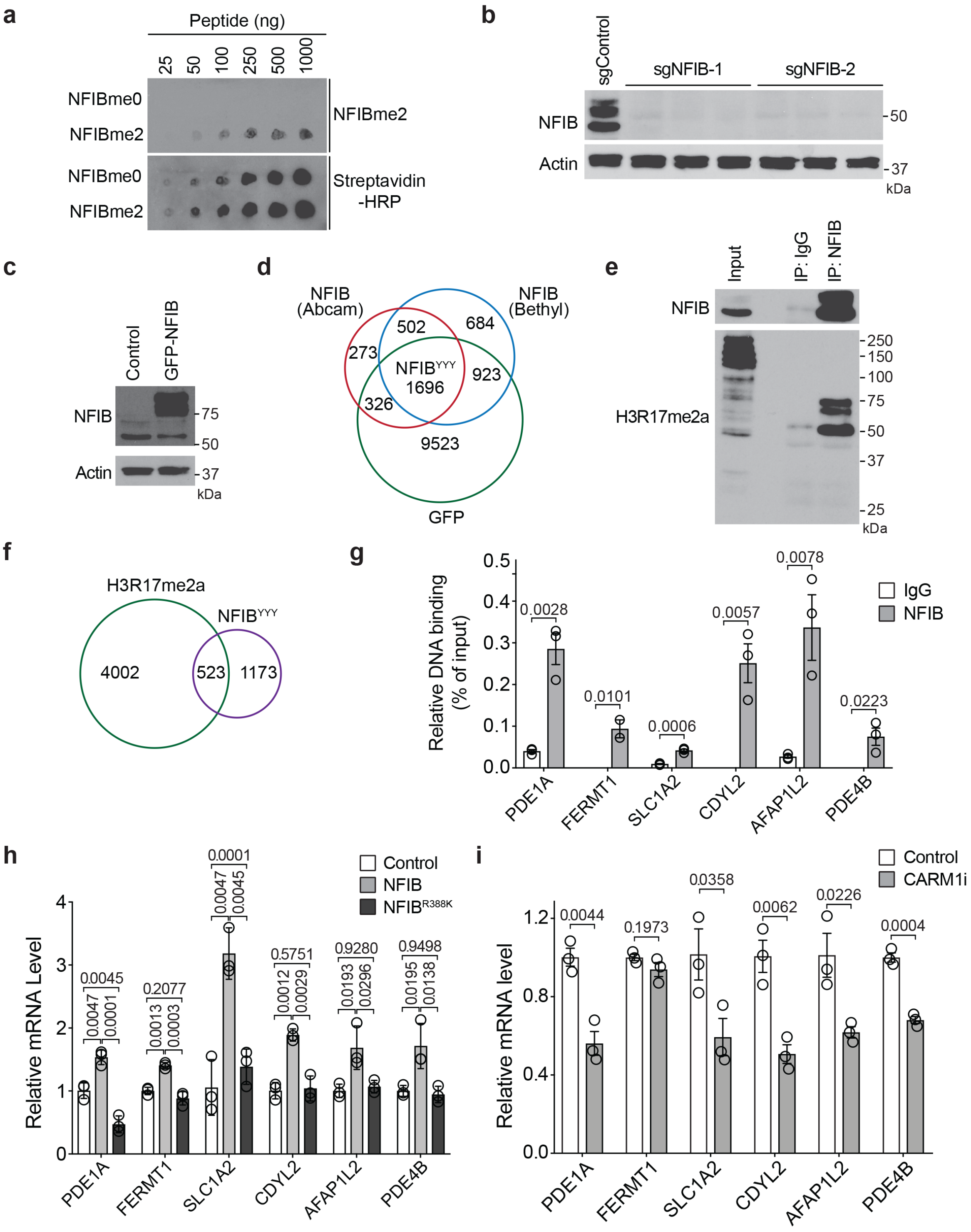

Supplementary Fig. 1 | Methylation of NFIB Is Regulating the Expression of NFIB Target Genes (related to Fig. 1). **a**, Dot blot showing the specific recognition of methyl-NFIB peptide by the NFIBme2a antibody made in house. **b**, Single-colony NFIB knockout cell lines were generated using CRISPR-Cas9 strategy in HeLa cells. The protein levels of NFIB were detected by Western blotting. **c**, Western blot showing the protein levels of NFIB in parent HeLa cells and HeLa cells stably expressing GFP-NFIB. **d**, Three individual ChIP-seq datasets were generated by immunoprecipitating NFIB with 2 independent NFIB antibodies from HeLa cells and immunoprecipitating GFP-NFIB from a HeLa cell line stably overexpressing GFP-NFIB. The Venn diagram shows the overlap of the ChIP-seq peaks from these 3 datasets. **e**, Endogenous NFIB proteins from HeLa cells were immunoprecipitated by the Bethyl NFIB antibody and tested for arginine methylation using the H3R17me2a antibody. **f**, Venn diagram showing the overlap of $NFIB^{YYY}$ peaks and ChIP-seq peaks using the H3R17me2a antibody. $NFIB^{YYY}$, common peaks in the 3 NFIB ChIP-seq datasets. **g**, Validation of NFIB binding to the indicated genes by ChIP-qPCR. **h**, Relative expression levels of indicated genes in HeLa cells ectopically expressing NFIB WT or R388K mutant. Biological replicates were performed. **i**, Relative expression levels of indicated genes in HeLa cells treated with CARM1 inhibitor. Experiments in **g**, **h** & **i** were performed in biological triplicate. Data present in mean ± SEM; *P* values determined by two-tailed student's t-test. All the blots are shown as representative data from three independent experiments.

Supplementary Figure 2

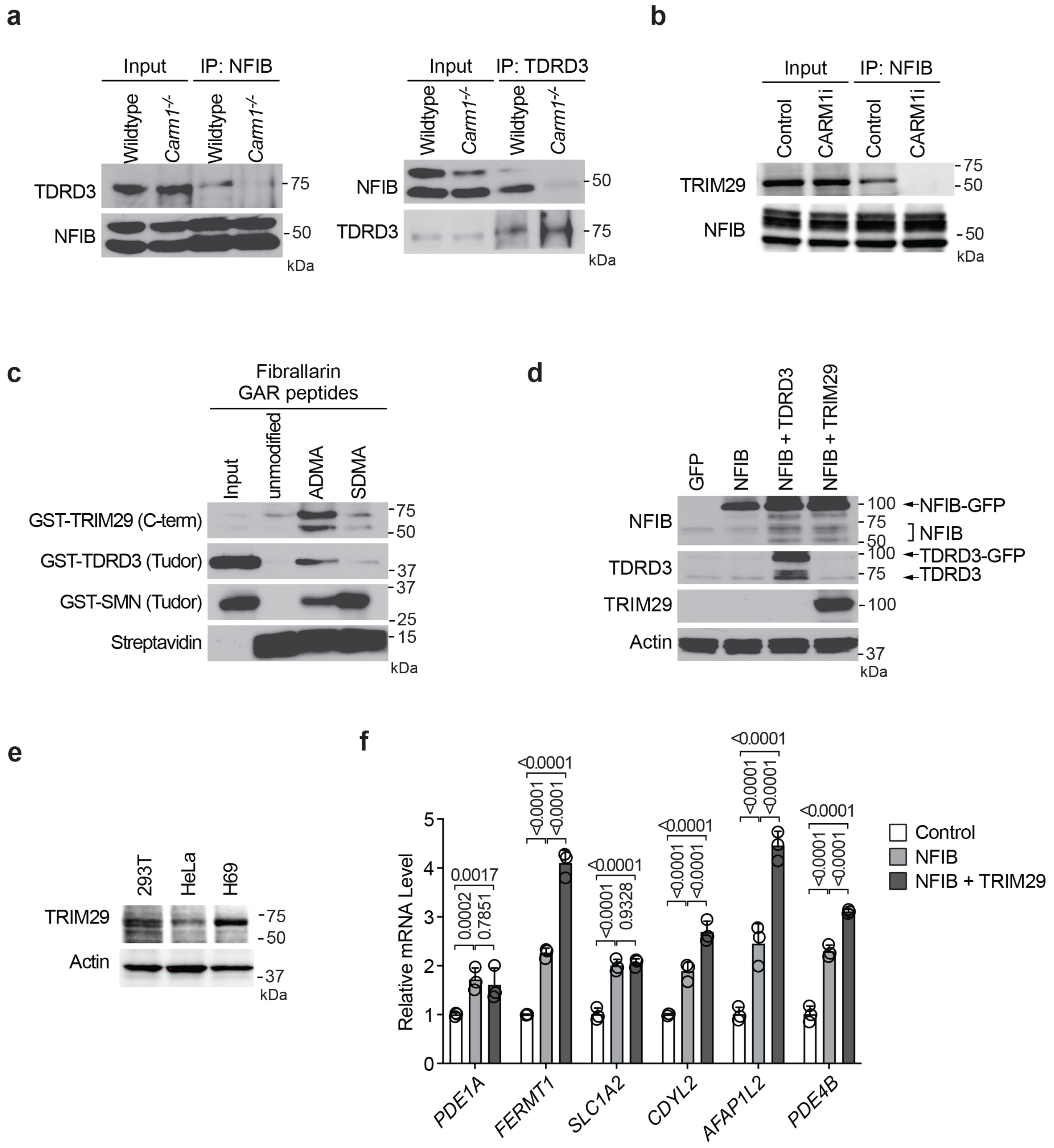

Supplementary Fig. 2 | TDRD3 and TRIM29 preferentially bind to ADMA marks (related to Fig. 2). **a**, Reciprocal co-immunoprecipitations of NFIB and TDRD3 in CARM1 WT or KO MEFs. **b**, H69 cells were treated with DMSO or 1 μM TP-064 for 2 days. Then the cell lysates were prepared, and co-IP was performed using the anti-NFIB antibody, followed by Western blot using the antibodies against NFIB or TRIM29. **c**, A series of biotin-labeled Fibrillarin GAR motif peptides (unmodified, ADMA, or SDMA modified) were incubated with GST-tagged TRIM29, TDRD3, or SMN domains. GST-tagged proteins binding to those peptides were enriched using Streptavidin-conjugated beads, followed by detection with GST antibody. **d**, 293T cells were co-transfected with GFAP-luc, together with GFP control, NFIB expressing vectors alone, or NFIB plus TDRD3 or TRIM29 constructs. Overexpression of NFIB, TDRD3, or TRIM29 was detected by Western blotting, serving as a control for **Fig. 2i**. **e**, TRIM29 protein levels in 293T, HeLa and H69 cells determined by Western blot using the TRIM29 antibody. **f**, HeLa cells were transfected with GFP control, GFP-NFIB, or GFP-NFIB plus FLAG-TRIM29 plasmids. The mRNA levels of indicated NFIB target genes were determined by RT-qPCR. Data present in mean ± SEM; *P* values determined by two-tailed student's t-test. All experiments were performed in biological triplicate.

Supplementary Figure 3

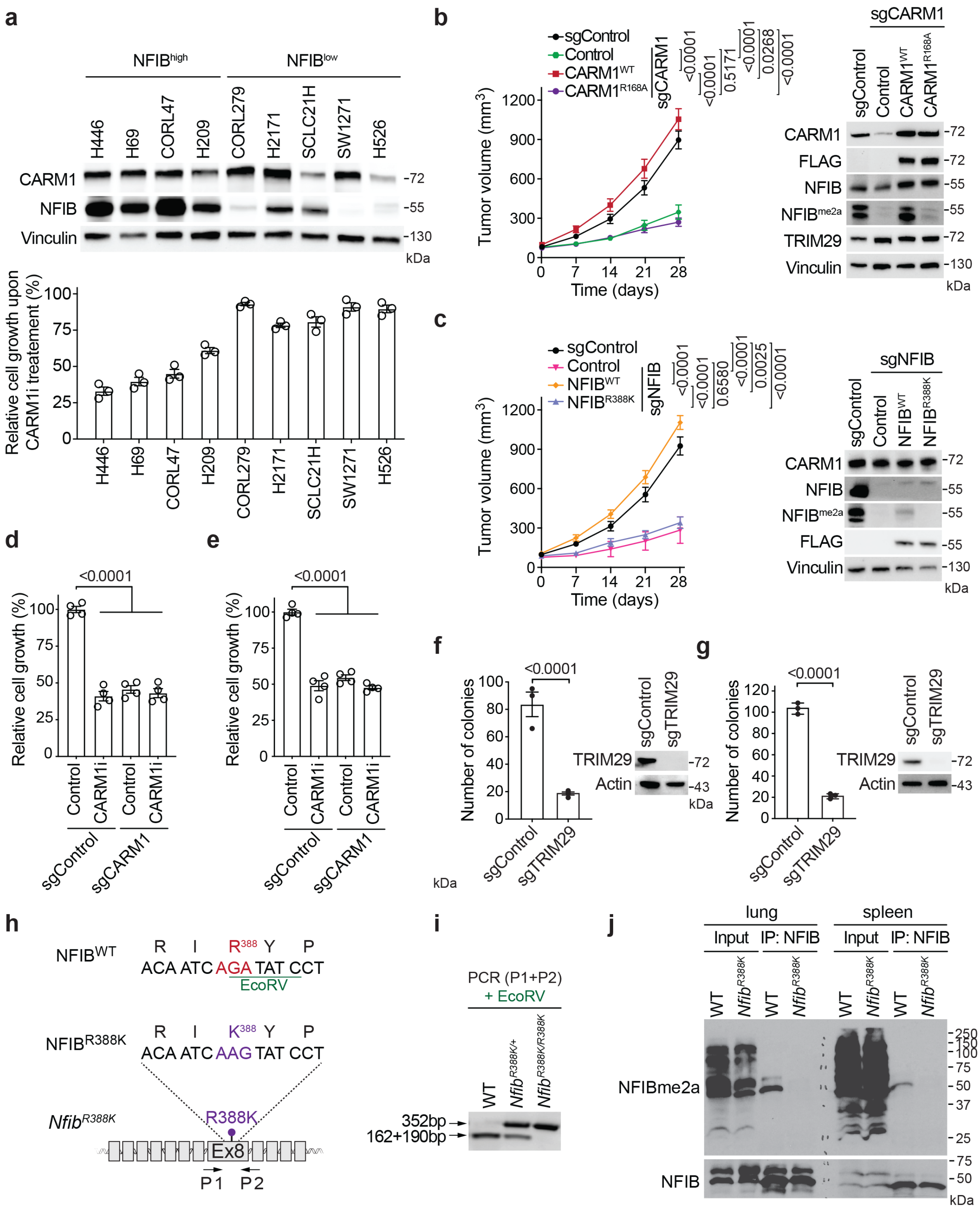

Supplementary Fig. 3 | Characterization of the CARM1-NFIB-TRIM29 pathway in SCLC cell (related to Fig. 3).

a, Immunoblot analysis of CARM1 and NFIB in cell lysates from indicated human SCLC cell lines. Vinculin shown as a loading control. NFIB expression correlates with cancer cells sensitivity to CARM1 inhibitor (TP-064), and these growth experiments were performed in biological triplicate. **b**, Complementation of sgRNA CARM1 depleted CORL47 SCLC cells with WT or R168A mutant CARM1 shows that CARM1 catalytic activity is required for SCLC cell growth *in vivo*. Tumor volume quantification of CORL47 xenografts in NSG mice treated as indicated (n=8 each group; *P* values were determined by two-way ANOVA with Tukey's testing for multiple comparisons, data represent mean ± SEM). Immunoblots with indicated antibodies of cell lysates depicted in the graph, asterisk indicate endogenous and arrowhead HA-tagged CARM1. **c**, Complementation of sgRNA NFIB depleted CORL47 SCLC cells with WT or R388K mutant NFIB shows that NFIB K388 methylation is required for SCLC cell growth *in vivo*. Tumor volume quantification of CORL47 xenografts in NSG mice treated as indicated (n=5 each group; *P* values were determined by two-way ANOVA with Tukey's testing for multiple comparisons, data represent mean ± SEM). Immunoblots with indicated antibodies of cell lysates depicted in the graph, asterisk indicate endogenous and arrowhead HA-tagged CARM1 and HA-tagged NFIB. Vinculin shown as a loading control. **d**-**e**, Analyses of (**d**) H69 and (**e**) CORL47 cells growth upon genetic and pharmacological CARM1 inhibition. sgCARM1 depletion as shown in Fig. 3d and Supplementary Fig. 3b, respectively. CARM1 inhibitor (TP-064). Experiments were performed in biological triplicate. Data present in mean ± SEM; *P* values determined by two-tailed student's test. **f**-**g**, Soft agar colony formation analyses of (**f**) H69 and (**g**) CORL47 cells with depletion of TRIM29. Immunoblot confirmation of TRIM28 depletion. Actin shown as a loading control. Data present in mean ± SEM; *P* values determined by two-tailed student's test. Experiments were performed in biological triplicate. **h**, Schematic showing the generation of the $\textit{Nfib}^{R388K}$ mouse model using CRISPR/Cas9 strategy. **i**, Representative genotyping for $\textit{Nfib}^{R388K}$ mice. After the PCR reaction, the DNA is subjected to EcoRV cleavage. DNA from $\textit{Nfib}^{R388K}$ homozygous mice is resistant to cleavage. **j**, Validation of the $\textit{Nfib}^{R388K}$ mouse model by NFIB immunoprecipitation and immunoblotting with the NFIBme2a antibody. Tissue samples were extracted from the lung and spleen of wildtype (WT) and $\textit{Nfib}^{R388K}$ homozygous mutant mice. Experiments were performed in triplicate.

Supplementary Figure 4

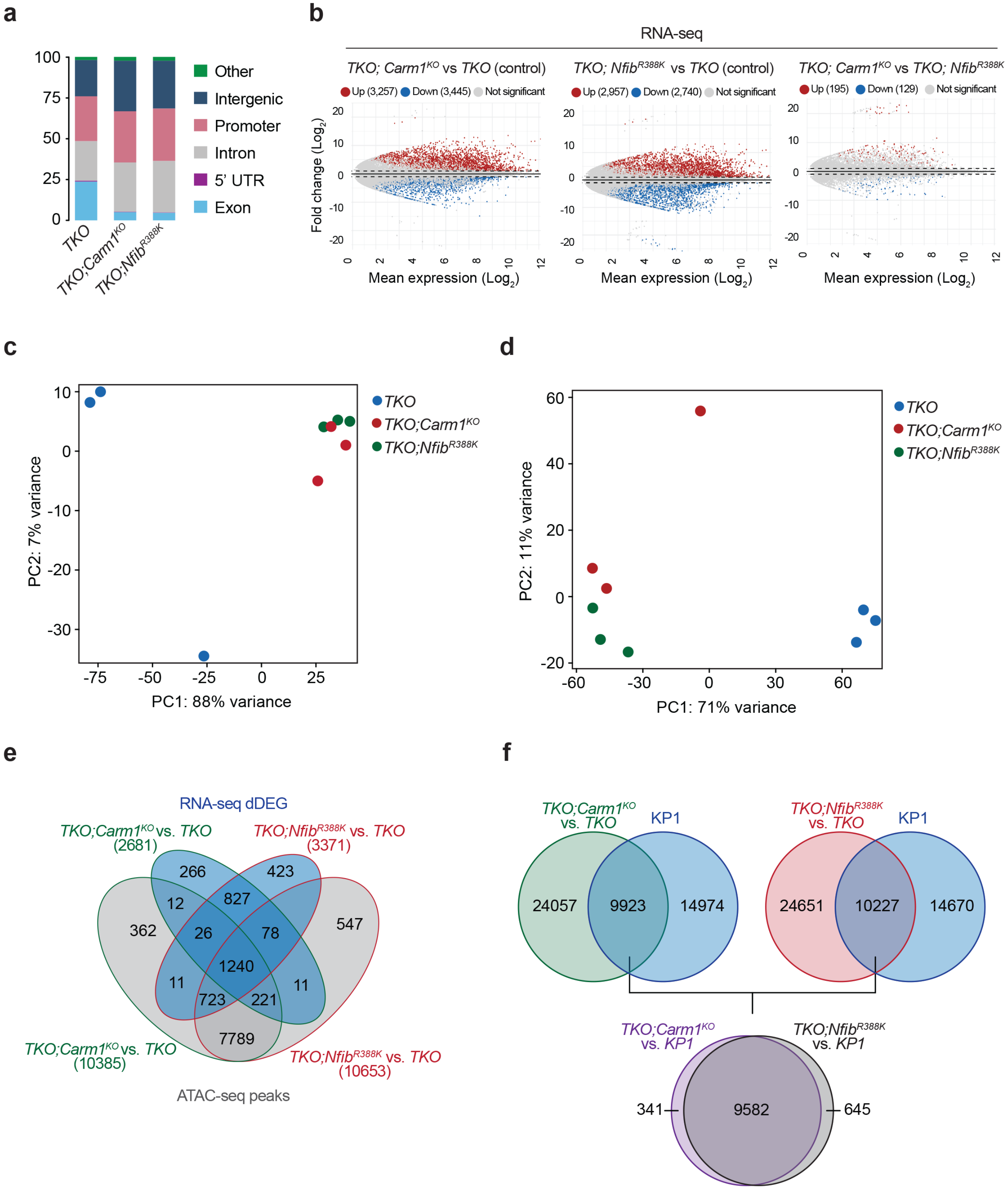

Supplementary Fig. 4 | The CARM1-NFIB axis (related to Fig. 4). **a**, ATAC-seq analysis of genomic regions with chromatin accessibility in indicated groups. **b**, RNA-seq analysis of deferentially expressed genes in indicated groups. **c**-**d**, The principle component analyses of (**c**) ATAC-seq and (**d**) RNA-seq results obtained from indicated samples. **e**, Venn diagram showing the number of genes with differential chromatin accessibility and expression levels in *TKO;Nfib*^{R388K} and *TKO;Carm1*^{KO} mutant mice relative to *TKO* (control) tumor biopsies the overlap of these targets between the two mutant mice. **f**, Venn diagram of NFIB ChIP-seq peaks obtained from murine KP1 cells[27] and the down-regulated ATAC-seq peaks in *TKO;Nfib*^{R388K} and *TKO;Carm1*^{KO} versus *TKO* control of which over 93% shared peaks between *TKO;Nfib*^{R388K} and *TKO;Carm1*^{KO}.

Supplementary Figure 5

a

Gene signatures (MSigDB identifier)	NES: *TKO;Nfib*^{R388K} vs. *TKO*	NES: *TKO;Carm1*^{KO} vs. *TKO*
HALLMARK_E2F targets (M1604)	-3.45	-3.71
HALLMARK_G2M checkpoint (M5901)	-3.04	-3.67
HALLMARK_MYC targets V1 (M1610)	-2.78	-1.91
HALLMARK_Mitotic spindle (M1613)	-1.99	-2.17
HALLMARK_DNA repair (M)	-1.61	-1.34
HALLMARK_Inflamatory response (M)	2.44	2.86
HALLMARK_Apoptosis (M1714)	1.17	1.55
Neuron fate specification (GO:0048665)	-2.35	-1.84
Chromatin modification (GO:0016568)	-2.12	-2.12
Chromatin organization (GO:0006325)	-2.06	-1.52
Cell cycle process (GO:0022402)	-2.04	-1.74
Chromatin remodeling (GO:0006338)	-2.01	-1.62
Neuron fate commintment (GO:0048663)	-1.96	-1.70
Chromatin silencing & neg.epigenetic reg of gene exp. (GO:0006342)	-1.80	-1.67
Positive reg. of immune response (GO:0050778)	2.66	2.28
Positive reg. of cell killing (GO:0031343)	2.05	1.47
Negative reg. of axon extension (GO:0030517)	1.37	1.33
Regulation of axon guidance (GO:1902667)	1.52	1.32

b

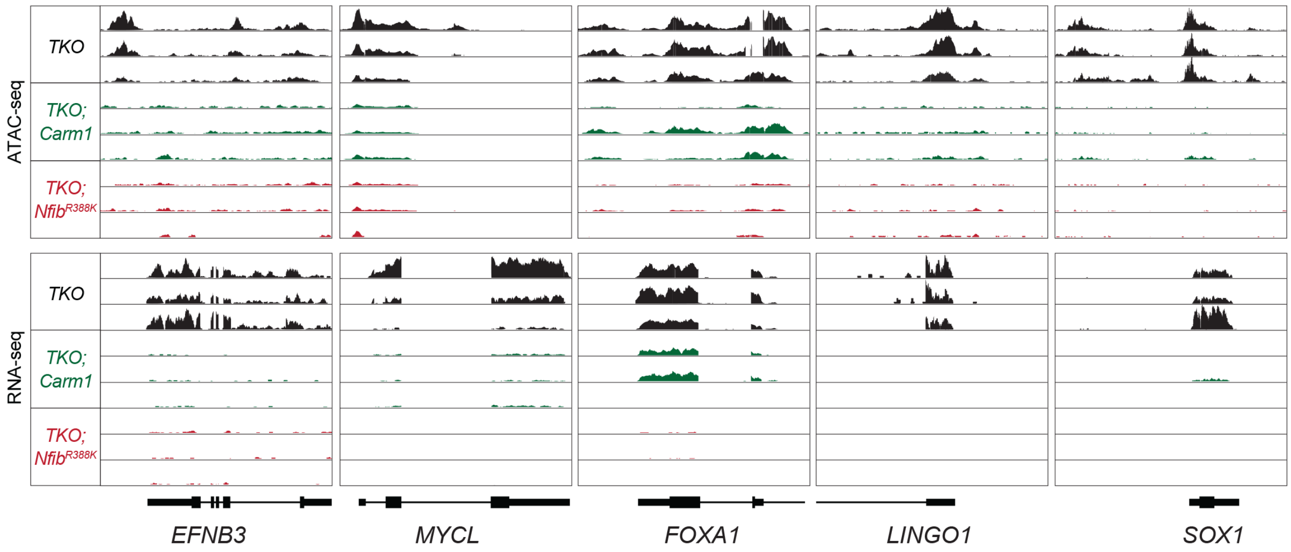

Supplementary Fig. 5 | The CARM1-NFIB axis (related to Fig. 4). **a**, GSEA of differentially expressed genes showing pathways significantly regulated in *TKO;Nfib*^{R388K} and *TKO;Carm1*^{KO} mutant mice versus *TKO* (control) tumor biopsies. The top gene sets identified in the GSEA analysis show high overlap. Normalized enrichment scores (NES) and false discovery rate (FDR) are provided (detailed statistics description in Methods). **b**, Integrative Genomic View (IGV) of ATAC-seq and RNA-seq signals on the indicated genes and conditions. Arrow indicates the direction of gene transcription. The tracks for each of the 9 independent tumors analyzed are shown.

Supplementary Table 1

**Primers used for RT-qPCR**	
PDE1A-F	AATGTGGCAGCGCCTGAAAGGA
PDE1A-R	CTTCCAGCACAGATGCCGCATA
FERMT1-F	CCAACTCTATGAGCAAGCCAGG
FERMT1-R	CCTGTGTTTCAGCAGACAACGAC
SLC1A2-F	TGCCAACAGAGGACATCAGCCT
SLC1A2-R	CAGCTCAGACTTGGAGAGGTGA
CDYL2-F	CGCCAGAATGAAAGCAACTGTCG
CDYL2-R	GTCGTCTGTGGCTGCGTTGCA
AFAP1L2-F	GCCACCCAAGATGATTCCAGA
AFAP1L2-R	GCCGTCCTCTTCATCGTAGG
PDE4B-F	TTCTCCACGCAGTTCACCAA
PDE4B-R	AAGGTGGCGTGAAGTACCAG
GAPDH-F	CATCACTGCCACCCAGAAGACTG
GAPDH-R	ATGCCAGTGAGCTTCCCGTTCAG
**primers used for ChIP-qPCR**	
PDE4B-ChIP-F	TCCGTGTTGACTCCACTTCACAT
PDE4B-ChIP-R	TCTGCTACAGTATAAACTTTTCC
PDE1A-ChIP-F	GACTACATGGGTTACTATGGTAT
PDE1A-ChIP-R	ACTTGAAATCTCACTTGCTT
AFAP1L2-ChIP-F	GCTTTTACATTTTTAAATGACTGAG
AFAP1L2-ChIP-R	TGCTCTAGAATTGGGGCTG
SLC1A2-ChIP-F	GCCTGCTTCTGATGTTGAGTGA
SLC1A2-ChIP-R	GCTTGGCCTTGGGATGGTGA
CDYL2-ChIP-F	GGCTTTGCCCTATACTAACATTC
CDYL2-ChIP-R	AACTGCTTTACTTGAGTTTATTGA
FERMT1-ChIP-F	GAAGAGACAGTCAAGGGTGCT
FERMT1-ChIP-R	CAATAAAAACAGAGATCAGAAGATG
**Primers used for genotyping**	
Nfib-geno-F	TCTCCAGTGTTACCGCGTTG
Nfib-geno-R	GCCTGGCTAAGCTCTCACTT
**Primers used for plasmid construction**	

FLAG-TRIM29 Fwd	GAGCGGCCGCAATGGAAGCTGCAGATGCCTCC
FLAG-TRIM29 Rev	GCGTCGACTCATGGGGCTTCGTTGGACCC
FLAG-TRIM29-N Rev	GCGTCGACGTCCCGGATGGGCTCGAGC
FLAG-TRIM29-CC Fwd	GAGCGGCCGCAGTGGAGGAGGCCAAGGCCGAG
FLAG-TRIM29-CC Rev	GCGTCGACATCCATGATCACCTTCACTTGG
FLAG-TRIM29-C Fwd	GAGCGGCCGCAGCTCTGGATGAGAGAGCCAAG
GST-TRIM29 Fwd	GCGGATCCATGGAAGCTGCAGATGCCTCC
GST-TRIM29 Rev	GCCTCGAGTCATGGGGCTTCGTTGGACCC
GST-TRIM29-C Fwd	GCGGATCCGCTCTGGATGAGAGAGCCAAG
GST-TRIM29-ΔC Rev	GAGCGGCCGCATCCATGATCACCTTCACTTGG
Mutagenesis NFIBR388K Fwd	GATTCAGGTGAGGAGGATATTTGATTGTTGGATGAGAGAAG
Mutagenesis NFIBR388K Rev	CTTCTCTCATCCAACAATCAAATATCCTCCTCACCTGAATC
Mutagenesis NFICR395K Fwd	AGATGTGGCGGGTACTTGATGGCCGTATGGGG
Mutagenesis NFICR395K Rev	CCCCATACGGCCATCAAGTACCCGCCACATCT
**sgRNA related oligos**	
sgRNA non-targeting control	CTTCGAAATGTCCGTTCGGT
sgRNA CARM1	ATCCAGTT CGCCACACCCAA
sgRNA NFIB for xenograft	TTCTCCCATCTGTCTCACTC
srRNA NFIB for HeLa cell	GATTGGATAAGACACAGCAC
sgRNA for NfibR388K mouse	TGAGGAGGATATCTGATTGTTGG
donor DNA for NfibR388K mouse	CGTTGCCATTTCCGACGCAAGCTATCCTTCCTCCGGCACCTTCCAGCT ACTTCTCTCATCCAACAATCAAGTATCCTCCTCACCTGAATCCTCAGG ATACTCTGAAGAACTACGTACCTTCTTATGACCCATCCAG

Source Data for Supplementary Figures

Fig. S1a

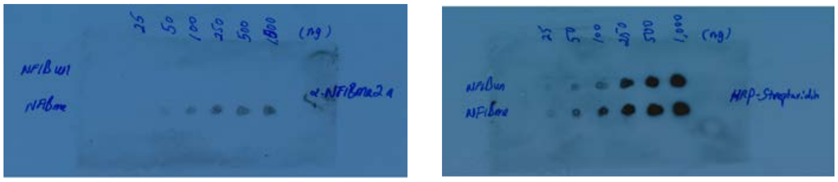

Fig. S1b

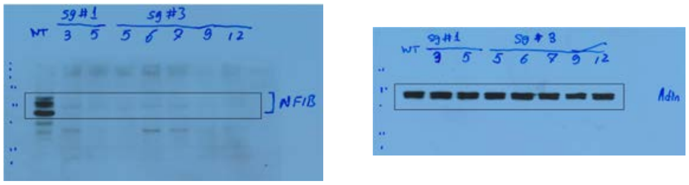

Fig. S1c

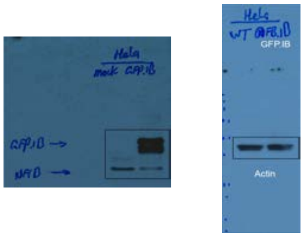

Fig. S1d
see Suppl Table S2A

Fig. S1e

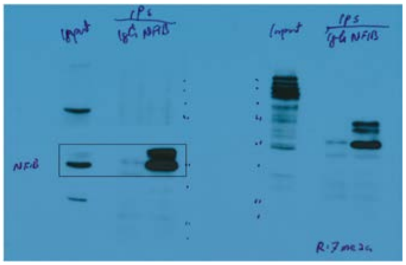

Fig. S1f
see Suppl Table S2B

Fig. S1g
relative DNA binding

	IgG			αNFIB		
PDE1A	0.040735	0.030731	0.042573	0.299646	0.312075	0.200195
FERMT1	n.d.	n.d.	n.d.	n.d.	0.109508	0.068487
SLC1A2	0.007046	0.010491	0.009632	0.044066	0.034003	0.041071
CDYL2	n.d.	n.d.	n.d.	0.256148	0.304249	0.154497
AFAP1L2	0.031996	0.020739	0.023733	0.177157	0.42922	0.351572
PDE4B	n.d.	n.d.	n.d.	0.076019	0.034965	0.10252

Fig. S1h
relative mRNA level

	GFP			NFIB-WT			NFIB-R388K		
PDE1A	1.081512	1.07187	0.862634	1.581904	1.403362	1.619784	0.61892	0.362846	0.434171
FERMT1	0.934673	1.042544	1.026233	1.357441	1.448227	1.413534	0.959487	0.76246	0.927225
SLC1A2	0.708435	1.55309	0.908873	2.90951	3.965356	2.98863	1.474737	1.61154	1.07101
CDYL2	1.052991	1.106539	0.85824	1.801576	1.878559	1.994005	0.970052	1.264101	0.889032
AFAP1L2	0.976456	1.121424	0.913224	1.527448	2.583252	1.451264	1.052532	1.175097	0.979675
PDE4B	0.970966	1.099195	0.93696	2.129498	1.505211	1.510059	1.084861	0.923103	0.829377

Fig. S1i
relative mRNA level

	DMSO			TP-064		
PDE1A	0.994388	0.9271	1.084719	0.480746	0.680115	0.523578
FERMT1	0.997881	1.029685	0.973234	0.953458	0.993142	0.870696
SLC1A2	1.263335	0.965633	0.819728	0.519723	0.478624	0.782563
CDYL2	0.862471	1.010751	1.147126	0.594656	0.494838	0.431646
AFAP1L2	0.94025	1.231707	0.863473	0.618045	0.66775	0.568319
PDE4B	1.045242	0.990511	0.965881	0.649144	0.683403	0.710054

Fig. S2a

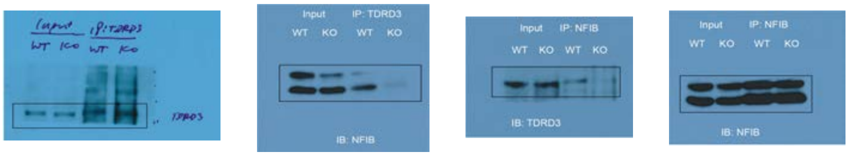

Fig. S2b

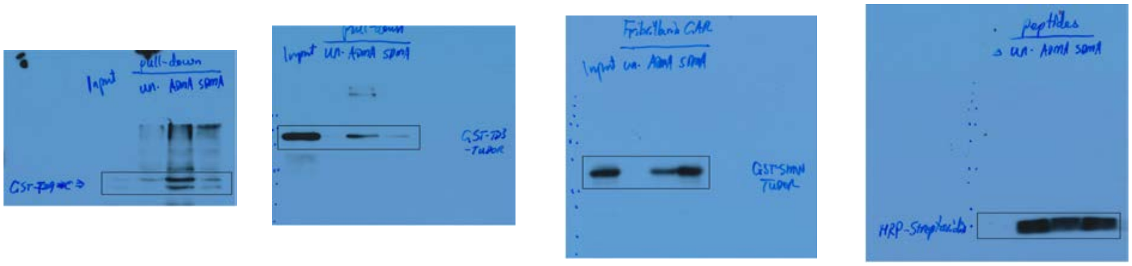

Fig. S2c

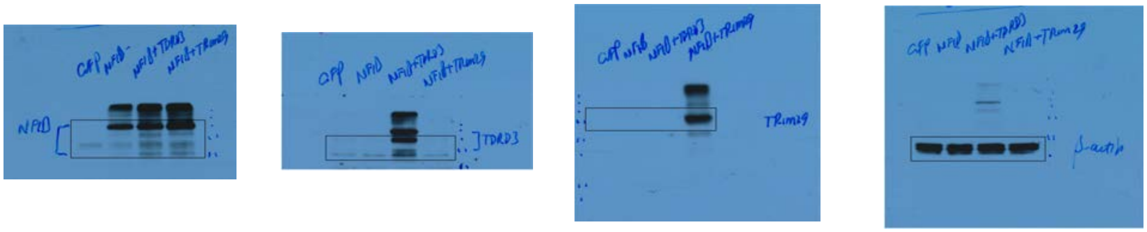

Fig. S2d

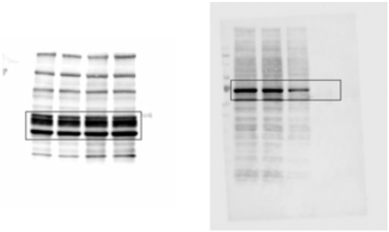

Fig. S2e

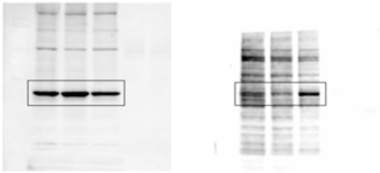

Fig. S2f

mRNA level for TRIM29-NFIB targets

	GFP			NFIB			NFIB+TRIM29		
PDE1A	1.030254	1.012555	0.958599	1.663244	1.975202	1.507335	2.005553	1.36604	1.451958
FERMT1	1.003457	0.988584	1.008063	2.300645	2.333072	2.146089	4.28016	4.214153	3.798192
SLC1A2	0.962967	0.901688	1.151681	2.144453	1.966036	1.893748	1.963083	2.119147	2.094295
CDYL2	0.974284	1.011587	1.014638	2.009616	1.975283	1.672128	2.943312	2.602999	2.516643
AFAP1L2	1.167698	0.888022	0.964375	2.779194	1.99262	2.584109	4.145846	4.480522	4.735992
PDE4B	1.177155	0.851951	0.99713	2.253699	2.434057	2.186833	3.14978	3.066976	3.107432

Fig. S3a

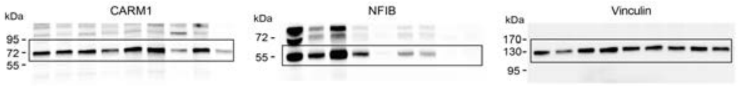

Fig. S3b

Day	Control					sgCARM1					sgCARM1+CARM1[WT]					sgCARM1+CARM1[R168A]				
	Xenograft tumor volume (mm^3)																			
0	101	98	90	120	92	84	90	109	91	115	125	118	116	106	120	81	90	92	95	102
7	200	181	129	229	168	92	114	128	120	169	231	141	244	282	280	102	121	100	134	150
14	310	311	220	440	298	124	146	185	151	218	488	232	439	476	480	121	173	150	198	214
21	640	550	400	710	482	221	211	304	197	401	791	429	678	820	800	157	237	200	290	303
28	905	927	740	1168	885	303	304	488	234	511	1191	802	1187	1240	1008	204	291	241	338	380

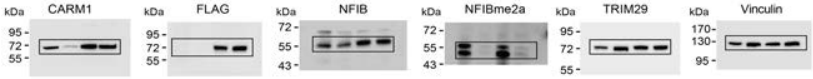

Fig. S3c

Day	Control					sgNFIB					sgNFIB+NFIB[WT]					sgNFIB+NFIB[R388K]				
	Xenograft tumor volume (mm^3)																			
0	101	98	90	120	92	95	88	79	67	71	120	110	109	114	95	98	90	81	97	84
7	200	181	129	229	168	119	104	82	70	90	280	225	189	282	160	125	111	111	114	96
14	310	311	220	440	298	198	209	120	81	102	455	405	392	488	300	148	192	211	290	124
21	640	550	400	710	482	301	246	187	122	164	614	687	610	881	650	281	252	325	260	141
28	905	927	740	1168	885	428	332	255	157	261	1111	1103	910	1202	1191	371	349	420	401	173

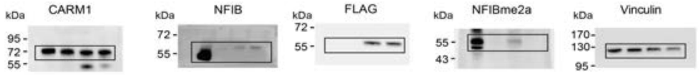

Fig. S3d

	Percent of viable cells (control 100%)			
sgControl+vehicle	104.62	102.46	96.97	95.95
sgControl+CARM1i	38.17	48.89	33.91	43.08
sgCARM1+vehicle	43.12	50.5	49.28	39.61
sgCARM1+CARM1i	50.75	37.49	45.15	38.92

Fig. S3e

	Percent of viable cells (control 100%)			
sgControl+vehicle	99.41	96.28	98.73	105.58
sgControl+CARM1i	48.59	55.67	52.1	39.48
sgCARM1+vehicle	51.96	50.43	57.06	58.7
sgCARM1+CARM1i	50.94	47.15	44.68	47.76

Fig. S3f

	Number of colonies		
sgControl	68	91	93
sgTRIM29	21	19	17

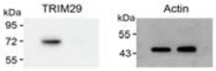

Fig. S3g

	Number of colonies		
sgControl	108	102	96
sgTRIM29	22	21	20

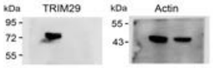

Fig. S3h

N/A

Fig. S3i

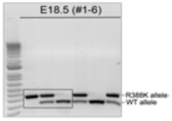

Fig. S3j

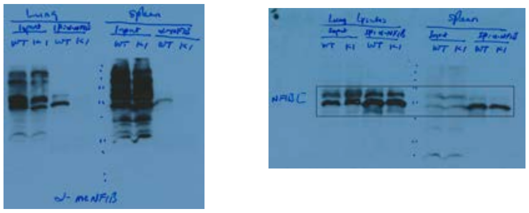

Fig. S4 & S5

See GEO accession: GSE195843
