# Supplementary material for: The NFIB/CARM1 partnership is a driver in preclinical models of small cell lung cancer
Source: Nat Commun. 2023 Jan 23;14:363. doi: 10.1038/s41467-023-35864-y (PMC9870865; doi:10.1038/s41467-023-35864-y)
Supplement: Supplementary file 2 — Description of Additional Supplementary Files [file 41467_2023_35864_MOESM2_ESM.pdf]

Description of Additional Supplementary Files

File Name: Supplementary Data 1
Description: Identification of CARM1 substrates

File Name: Supplementary Data 2
Description: NFIB targets

File Name: Supplementary Data 3
Description: Screening for NFIBme2a readers

File Name: Supplementary Data 4
Description: TRIM29 binding partners
