# Supplementary material for: The NFIB/CARM1 partnership is a driver in preclinical models of small cell lung cancer
Source: Nat Commun. 2023 Jan 23;14:363. doi: 10.1038/s41467-023-35864-y (PMC9870865; doi:10.1038/s41467-023-35864-y)
Supplement: Supplementary file 7 — Reporting Summary [file 41467_2023_35864_MOESM7_ESM.pdf]

nature portfolio

Corresponding author(s): Mark T. Bedford

Last updated by author(s): Dec 4, 2022

Reporting Summary

Nature Portfolio wishes to improve the reproducibility of the work that we publish. This form provides structure for consistency and transparency in reporting. For further information on Nature Portfolio policies, see our Editorial Policies and the Editorial Policy Checklist.

Statistics

For all statistical analyses, confirm that the following items are present in the figure legend, table legend, main text, or Methods section.

n/a	Confirmed	
☐	☒	The exact sample size (*n*) for each experimental group/condition, given as a discrete number and unit of measurement
☐	☒	A statement on whether measurements were taken from distinct samples or whether the same sample was measured repeatedly
☐	☒	The statistical test(s) used AND whether they are one- or two-sided<br>*Only common tests should be described solely by name; describe more complex techniques in the Methods section.*
☒	☐	A description of all covariates tested
☐	☒	A description of any assumptions or corrections, such as tests of normality and adjustment for multiple comparisons
☐	☒	A full description of the statistical parameters including central tendency (e.g. means) or other basic estimates (e.g. regression coefficient) AND variation (e.g. standard deviation) or associated estimates of uncertainty (e.g. confidence intervals)
☐	☒	For null hypothesis testing, the test statistic (e.g. *F*, *t*, *r*) with confidence intervals, effect sizes, degrees of freedom and *P* value noted<br>*Give P values as exact values whenever suitable.*
☒	☐	For Bayesian analysis, information on the choice of priors and Markov chain Monte Carlo settings
☒	☐	For hierarchical and complex designs, identification of the appropriate level for tests and full reporting of outcomes
☐	☒	Estimates of effect sizes (e.g. Cohen's *d*, Pearson's *r*), indicating how they were calculated

Our web collection on statistics for biologists contains articles on many of the points above.

Software and code

Policy information about availability of computer code

Data collection	PreciPoint M8 ViewPoint was used to aquire the histological images.
Data analysis	ImageJ2/FIJI was used to analyze the images; Prism 8 and Excel for Mac 2016 were used to do the statistical analysis.<br>Proteome Discoverer software Version 2.1 was used to assign peptide sequences from mass spectrum. Scaffold 5 was used to analyze the proteomics data.

For manuscripts utilizing custom algorithms or software that are central to the research but not yet described in published literature, software must be made available to editors and reviewers. We strongly encourage code deposition in a community repository (e.g. GitHub). See the Nature Portfolio guidelines for submitting code & software for further information.

Data

Policy information about availability of data

All manuscripts must include a data availability statement. This statement should provide the following information, where applicable:

- Accession codes, unique identifiers, or web links for publicly available datasets
- A description of any restrictions on data availability
- For clinical datasets or third party data, please ensure that the statement adheres to our policy

The RNA-seq and ATAC-seq data generated in this study has been deposited in the GEO database under accession code GSE195843. The mass spectrometry proteomics data have deposited to the ProteomeXchange via PRIDE with the identifier PXD038237, and via MASSive with the identifier MSV000090746.

Human research participants

Policy information about studies involving human research participants and Sex and Gender in Research.

Reporting on sex and gender	*Use the terms sex (biological attribute) and gender (shaped by social and cultural circumstances) carefully in order to avoid confusing both terms. Indicate if findings apply to only one sex or gender; describe whether sex and gender were considered in study design whether sex and/or gender was determined based on self-reporting or assigned and methods used. Provide in the source data disaggregated sex and gender data where this information has been collected, and consent has been obtained for sharing of individual-level data; provide overall numbers in this Reporting Summary. Please state if this information has not been collected. Report sex- and gender-based analyses where performed, justify reasons for lack of sex- and gender-based analysis.*
Population characteristics	*Describe the covariate-relevant population characteristics of the human research participants (e.g. age, genotypic information, past and current diagnosis and treatment categories). If you filled out the behavioural & social sciences study design questions and have nothing to add here, write "See above."*
Recruitment	*Describe how participants were recruited. Outline any potential self-selection bias or other biases that may be present and how these are likely to impact results.*
Ethics oversight	*Identify the organization(s) that approved the study protocol.*

Note that full information on the approval of the study protocol must also be provided in the manuscript.

Field-specific reporting

Please select the one below that is the best fit for your research. If you are not sure, read the appropriate sections before making your selection.

☒ Life sciences ☐ Behavioural & social sciences ☐ Ecological, evolutionary & environmental sciences

For a reference copy of the document with all sections, see nature.com/documents/nr-reporting-summary-flat.pdf

Life sciences study design

All studies must disclose on these points even when the disclosure is negative.

Sample size	For cell line experiments, at least 3 independent samples were used for each analysis; for the analysis on GEMM mouse models, more than 6 mice were used; for xenografts studies, 8 mice were used in each group; for PDX models, 8 mice were used for each group; and for patient sample analysis, a total of 32 samples were included.
Data exclusions	No data were excluded from analysis
Replication	Except for the in vivo studies, all experiments presented in this study were performed at least 3 times under independent experimental conditions. All attempts at replication have been successful.
Randomization	For the in vivo studies, animals were assigned randomly to experimental and control groups. Other experiments were not randomized but were usually performed in different format (e.g., 6-well plates vs. 24-well plates).
Blinding	Blinding was performed in this study because it requires mixing of wild-type and mutants and this could lead to the risk of mislabeling.

Reporting for specific materials, systems and methods

We require information from authors about some types of materials, experimental systems and methods used in many studies. Here, indicate whether each material, system or method listed is relevant to your study. If you are not sure if a list item applies to your research, read the appropriate section before selecting a response.

Materials & experimental systems

n/a	Involved in the study
☐	☒ Antibodies
☐	☒ Eukaryotic cell lines
☒	☐ Palaeontology and archaeology
☐	☒ Animals and other organisms
☒	☐ Clinical data
☒	☐ Dual use research of concern

Methods

n/a	Involved in the study
☐	☒ ChIP-seq
☒	☐ Flow cytometry
☒	☐ MRI-based neuroimaging

Antibodies

Antibodies used

CARM1, Bethyl, Cat# A300-421A
NFIB (ab), Abeam, Cat# ab186738
NFIB (be), Bethyl, Cat# A303-566A
NFIB R388me2a, made in house (This paper)
TRIM29, Santa Cruz Biotechnology, Cat# sc-166718
TDRD3, Millipore, Cat# MABE1042
GFP (WB), Santa Cruz Biotechnology, Cat# sc-9996
GFP (IP), Invitrogen, Cat# A6455
GST, made in house
Actin, Sigma-Aldrich, Cat# A1978
pan-ADMA, made in house (Wang et al., 2021)
pan-SDMA, made in house (Wang et al., 2021)
H3R17me2a, Millipore, Cat# 07-214
pH3SerlO, Millipore, Cat# 06-570
Vinculin, Cell Signaling, Technology Cat# 13901
FLAG, Sigma-Aldrich, Cat# F1804
MYCL, Thermo Fisher Scientific, Cat# PA5109998
EFNB3, Thermo Fisher Scientific, Cat# 34-3600
LINGO1/2, Cell Signaling Technology, Cat# 49389
FOXA1, Thermo Fisher Scientific, Cat# MAS-32556
SOX1, Thermo Fisher Scientific, Cat# MAS-32447
Peroxidase AffiniPure Donkey Anti-Mouse lgG Jackson lmmunoResearch Cat# 715-035-151
Peroxidase AffiniPure Donkey Anti-Rabbit lgG Jackson lmmunoResearch Cat# 711-035-152

Validation

Except for the validation data available on the antibody websites, we have shown validations for some key antibodies we used: Anti-CARM1 antibody is validated using CARMl-KO MEF cells; this antibody works for both human and mouse CARM1; it works for Western blots and IP.
Anti-NFIB antibodies were validated for Western blots using NFIB-KO HeLa cells; these antibodies recognize both human and mouse NFIB; they were both also validated for immunoprecipitation as well.
Anti-pan-ADMA/-SDMA antibodies were validated in PRMT1 and PRMT5 inducible KO MEFs as well as inhibitors to PRMT1 and PRMT5;
Anti-NFIBme2a antibody was validated with control and NFIBme2a peptides, NFIB KO cells and cells treated with CARMl inhibitor.
Anti-TRIM29 antibody was validated by TRIM29 overexpression; it can recognize both human and mouse TRIM29 by Western blots.

Eukaryotic cell lines

Policy information about cell lines and Sex and Gender in Research

Cell line source(s)

293T ATCC CRL-3216
HeLa ATCC CCL2
H69 ATCC HTB-119
CORL47 Sigma-Aldrich 92031915
H446 ATCC HTB-171
H209 ATCC HTB-172
CORL279 Sigma-Aldrich 96020724
H2171 ATCC CRL-5929
SCLC21H Accegen ABC-TC605S
SW1271 ATCC CRL-2177
H526 ATCC CRL-5811

Authentication

Cell lines were freshly purchase for studies presented in this manuscript. When needed cell identity was confirmed using the "Cytogenetics and Cell Authentication Core", at MD Anderson Cancer Center.

Mycoplasma contamination

Cell lines in culture are routinely tested for mycoplasma contamination in the Bedford lab

Commonly misidentified lines
(See ICLAC register)

HeLa

Animals and other research organisms

Policy information about studies involving animals; ARRIVE guidelines recommended for reporting animal research, and Sex and Gender in Research

Laboratory animals	Mouse: p53 LoxP/LoxP, The Jackson Laboratories, Strain# 008462<br>Mouse: Rbl LoxP/LoxP, The Jackson Laboratories, Strain# 026563<br>Mouse: Rbl2LoxP/LoxP, The Jackson Laboratories, Strain# 008177<br>Mouse: Rosa26LSL-tdTomato, The Jackson Laboratories, Strain# 007914 Mouse: Carml LoxP/LoxP, (Yadav et al., 2003)<br>Mouse: NfibR388K, described in this paper<br>Mouse: NOD.SCID-IL2Rg-/- (NSG), The Jackson Laboratories ,Strain# 005557<br>Mouse: C57BL/6, The Jackson Laboratories, Strain# 000664<br>Mouse: FVB129/N, The Jackson Laboratories, Strain# 001800<br>The sex and age of the mice used are randomized.<br>We used the animals to study small cell lung cancer which is not affected by the gender of the animals, and sex was not considered in the study design.
Wild animals	No wild animals were used in this study.
Reporting on sex	N/A
Field-collected samples	No field-collected samples were used in this study.
Ethics oversight	Mouse handling and care followed the NIH Guide for Care and Use of Laboratory Animals. All animal procedures followed the guidelines of and were approved by the MDACC Institutional Animal Care and Use Committee (IACUC protocol 00001636, Pl: Mazur).

Note that full information on the approval of the study protocol must also be provided in the manuscript.

ChIP-seq

Data deposition

☒ Confirm that both raw and final processed data have been deposited in a public database such as GEO.

☒ Confirm that you have deposited or provided access to graph files (e.g. BED files) for the called peaks.

Data access links<br>*May remain private before publication.*	GEO accession number GSE219265.
Files in database submission	HeLa_NFIB_ab_1.peaks.txt<br>HeLa_NFIB_ab_2.peaks.txt<br>HeLa_NFIB_be_1.peaks.txt<br>HeLa_NFIB_be_2.peaks.txt<br>NFIB_GFP_1.peaks.txt<br>NFIB_GFP_2.peaks.txt<br>8-CHIP.peaks.txt<br>merged_HeLa_NFIB_ab.peaks.txt<br>merged_HeLa_NFIB_ab.peaks.txt<br>merged_HeLa_NFIB_be.peaks.txt<br>merged_HeLa_NFIB_be.peaks.txt<br>merged_NFIB_GFP.peaks.txt<br>merged_NFIB_GFP.peaks.txt<br>HeLa_Input_1_ATCACG_L001_R1_001.fastq.gz<br>HeLa_Input_2_TTAGGC_L001_R1_001.fastq.gz<br>HeLa_NFIB_ab_1_GATCAG_L001_R1_001.fastq.gz<br>HeLa_NFIB_ab_2_CCGTCC_L001_R1_001.fastq.gz<br>HeLa_NFIB_be_1_GTCCGC_L001_R1_001.fastq.gz<br>HeLa_NFIB_be_2_ATCACG_L002_R1_001.fastq.gz<br>NFIB_GFP_1_CCGTCC_L002_R1_001.fastq.gz<br>NFIB_GFP_2_GTCCGC_L002_R1_001.fastq.gz<br>NFIB_Input_1_TTAGGC_L002_R1_001.fastq.gz<br>NFIB_Input_2_GATCAG_L002_R1_001.fastq.gz<br>8-CHIP.fastq.gz<br>8-input.fastq.gz<br>HeLa_Input_1_ATCACG_L001_R1_002.fastq.gz<br>HeLa_Input_2_TTAGGC_L001_R1_002.fastq.gz<br>HeLa_NFIB_ab_1_GATCAG_L001_R1_002.fastq.gz<br>HeLa_NFIB_ab_2_CCGTCC_L001_R1_002.fastq.gz<br>HeLa_NFIB_be_1_GTCCGC_L001_R1_002.fastq.gz<br>HeLa_NFIB_be_2_ATCACG_L002_R1_002.fastq.gz<br>NFIB_GFP_1_CCGTCC_L002_R1_002.fastq.gz<br>NFIB_GFP_2_GTCCGC_L002_R1_002.fastq.gz<br>NFIB_Input_1_TTAGGC_L002_R1_002.fastq.gz

NFIB_Input_2_GATCAG_L002_R1_002.fastq.gz
HeLa_Input_1_ATCACG_L001_R1_003.fastq.gz
HeLa_Input_2_TTAGGC_L001_R1_003.fastq.gz
HeLa_NFIB_ab_1_GATCAG_L001_R1_003.fastq.gz
HeLa_NFIB_ab_2_CCGTCC_L001_R1_003.fastq.gz
HeLa_NFIB_be_1_GTCCGC_L001_R1_003.fastq.gz
HeLa_NFIB_be_2_ATCACG_L002_R1_003.fastq.gz
NFIB_GFP_1_CCGTCC_L002_R1_003.fastq.gz
NFIB_GFP_2_GTCCGC_L002_R1_003.fastq.gz
NFIB_Input_1_TTAGGC_L002_R1_003.fastq.gz
NFIB_Input_2_GATCAG_L002_R1_003.fastq.gz
HeLa_Input_1_ATCACG_L001_R1_004.fastq.gz
HeLa_Input_2_TTAGGC_L001_R1_004.fastq.gz
HeLa_NFIB_ab_1_GATCAG_L001_R1_004.fastq.gz
HeLa_NFIB_ab_2_CCGTCC_L001_R1_004.fastq.gz
HeLa_NFIB_be_1_GTCCGC_L001_R1_004.fastq.gz
HeLa_NFIB_be_2_ATCACG_L002_R1_004.fastq.gz
NFIB_GFP_1_CCGTCC_L002_R1_004.fastq.gz
NFIB_GFP_2_GTCCGC_L002_R1_004.fastq.gz
NFIB_Input_1_TTAGGC_L002_R1_004.fastq.gz
NFIB_Input_2_GATCAG_L002_R1_004.fastq.gz
HeLa_Input_1_ATCACG_L001_R1_005.fastq.gz
HeLa_Input_2_TTAGGC_L001_R1_005.fastq.gz
HeLa_NFIB_ab_1_GATCAG_L001_R1_005.fastq.gz
HeLa_NFIB_ab_2_CCGTCC_L001_R1_005.fastq.gz
HeLa_NFIB_be_1_GTCCGC_L001_R1_005.fastq.gz
HeLa_NFIB_be_2_ATCACG_L002_R1_005.fastq.gz
NFIB_GFP_1_CCGTCC_L002_R1_005.fastq.gz
NFIB_GFP_2_GTCCGC_L002_R1_005.fastq.gz
NFIB_Input_1_TTAGGC_L002_R1_005.fastq.gz
NFIB_Input_2_GATCAG_L002_R1_005.fastq.gz
HeLa_Input_1_ATCACG_L001_R1_006.fastq.gz
HeLa_Input_2_TTAGGC_L001_R1_006.fastq.gz
HeLa_NFIB_ab_1_GATCAG_L001_R1_006.fastq.gz
HeLa_NFIB_ab_2_CCGTCC_L001_R1_006.fastq.gz
HeLa_NFIB_be_1_GTCCGC_L001_R1_006.fastq.gz
HeLa_NFIB_be_2_ATCACG_L002_R1_006.fastq.gz
NFIB_GFP_1_CCGTCC_L002_R1_006.fastq.gz
NFIB_GFP_2_GTCCGC_L002_R1_006.fastq.gz
NFIB_Input_1_TTAGGC_L002_R1_006.fastq.gz
NFIB_Input_2_GATCAG_L002_R1_006.fastq.gz
HeLa_Input_1_ATCACG_L001_R1_007.fastq.gz
HeLa_Input_2_TTAGGC_L001_R1_007.fastq.gz
HeLa_NFIB_ab_1_GATCAG_L001_R1_007.fastq.gz
HeLa_NFIB_ab_2_CCGTCC_L001_R1_007.fastq.gz
HeLa_NFIB_be_1_GTCCGC_L001_R1_007.fastq.gz
HeLa_NFIB_be_2_ATCACG_L002_R1_007.fastq.gz
NFIB_GFP_1_CCGTCC_L002_R1_007.fastq.gz
NFIB_GFP_2_GTCCGC_L002_R1_007.fastq.gz
NFIB_Input_1_TTAGGC_L002_R1_007.fastq.gz
NFIB_Input_2_GATCAG_L002_R1_007.fastq.gz
HeLa_Input_1_ATCACG_L001_R1_008.fastq.gz
HeLa_Input_2_TTAGGC_L001_R1_008.fastq.gz
HeLa_NFIB_ab_1_GATCAG_L001_R1_008.fastq.gz
HeLa_NFIB_ab_2_CCGTCC_L001_R1_008.fastq.gz
HeLa_NFIB_be_1_GTCCGC_L001_R1_008.fastq.gz
NFIB_GFP_2_GTCCGC_L002_R1_008.fastq.gz
HeLa_Input_1_ATCACG_L001_R1_009.fastq.gz
HeLa_Input_2_TTAGGC_L001_R1_009.fastq.gz
NFIB_GFP_2_GTCCGC_L002_R1_009.fastq.gz

Genome browser session
(e.g. UCSC)

UCSC genome browser human genome assembly hg19: https://genome.ucsc.edu/cgi-bin/hgTracks?db=hg19

Methodology

Replicates

3 antibodies were used for NFIB peaks (GFP, NFIB-ab and NFIB-be) and each were represented by 2 replicates. 1 antibody was used for H3R17me2a peaks and it was done using 3 replicates.

Sequencing depth

26-33 million reads were generated for NFIB peaks per sample. 46 and 52 million reads were generated for H3R17me2a.

Antibodies

NFIB (ab), Abeam, Cat# ab186738 NFIB (be), Bethyl, Cat# A303-566A GFP (IP), lnvitrogen, Cat# A6455 H3R17me2a, Millipore, Cat# 07-214

Peak calling parameters

To obtain the NFIB peaks pulled down by each antibody, the two replicate samples were merged to a merged sample and peak

Peak calling parameters	calling was performed on both the merged sample and each individual replicate by MACS (Zhang et al., 2008) using total input DNA as the negative control. The window size was set as 300 bp and the P-value cutoff was set as lXlOe-5. The peaks overlapped with ENCODE blacklisted regions (Consortium, 2012) were removed.<br>The analysis for H3R17me2a, including mapping and peak calling, was the same as in (Cheng et al., 2018) (briefly, the same as NFIB, except the mapping was done using bowtie (version 0.12.8) (Langmead et al., 2009) and the P-value cutoff for peak calling was lXl0e-6 to control the empirical FDR below 0.05).
Data quality	For NFIB Chip-seq, 81-93% of the total reads were mapped to the human genome, while 68-78% of the total reads were uniquely mapped.<br>For H3R17me2a ChlP-seq, 95% and 97% of the total reads were mapped to the human genome, with 73% and 74% uniquely mapped.
Software	Sequenced DNA reads were mapped to the human genome hg19 using lllumina analysis pipeline CASAVA (version 1.8.2).
